# Sexually dimorphic metabolic responses mediated by CRF_2_ receptor during nutritional stress in mice

**DOI:** 10.1186/s13293-018-0208-4

**Published:** 2018-11-06

**Authors:** Sreenivasan Paruthiyil, Shin-ichiro Hagiwara, Keshav Kundassery, Aditi Bhargava

**Affiliations:** Department of Obstetrics and Gynecology, Center for reproductive Sciences, and the Osher Center for Integrative Medicine, University of California San Francisco, 513 Parnassus Ave., HSE1645, Box 0556, San Francisco, CA 94143-0556 USA

**Keywords:** Blood glucose, Dyslipidemia, Fat mass, Hepatic steatosis, Plasma insulin

## Abstract

**Background:**

Chronic stress is a major contributor in the development of metabolic syndrome and associated diseases, such as diabetes. High-fat diet (HFD) and sex are known modifiers of metabolic parameters. Peptide hormones corticotropin-releasing factor (CRF) and urocortins (UCN) mediate stress responses via activation and feedback to the hypothalamic-pituitary-adrenal (HPA) axis. UCN3 is a marker of pancreatic β-cell differentiation, and UCN2 is known to ameliorate glucose levels in mice rendered diabetic with HFD. CRF receptor 2 (CRF_2_) is the only known cognate receptor for UCN2/3. Here, we ascertained the role of CRF_2_ in glucose clearance, insulin sensitivity, and other parameters associated with metabolic syndrome in a mouse model of nutritional stress.

**Methods:**

Wild-type (WT) and Crhr2^−/−^ (null) mice of both sexes were fed either normal chow diet or HFD. After 8 weeks, blood glucose levels in response to glucose and insulin challenge were determined. Change in body and fat mass, plasma insulin, and lipid profile were assessed. Histological evaluation of liver sections was performed.

**Results:**

Here, we show that genotype (Crhr2), sex, and diet were all independent variables in the regulation of blood glucose levels, body and fat mass gain/redistribution, and insulin resistance. Surprisingly, CRF_2_-deficient mice (Crhr2^−/−^) male mice showed similarly impaired glucose clearance on HFD and chow. HFD-fed female Crhr2^−/−^ mice redistributed their fat depots that were distinct from wild-type females and male mice on either diet. Blood cholesterol and low-density lipoprotein (LDL) levels were elevated significantly in male Crhr2^−/−^ mice; female Crhr2^−/−^ mice were protected. Male, but not female Crhr2^−/−^ mice developed peripheral insulin resistance. HFD, but not chow-fed wild-type male mice developed hepatic macrovesicular steatosis. In contrast, livers of Crhr2^−/−^ male mice showed microvesicular steatosis on either diet, whereas livers of female mice on this 8-week HFD regimen did not develop steatosis.

**Conclusions:**

CRF_2_ receptor dysregulation is a sexually dimorphic risk factor in development of pre-diabetic and metabolic symptoms.

**Electronic supplementary material:**

The online version of this article (10.1186/s13293-018-0208-4) contains supplementary material, which is available to authorized users.

## Introduction

Stress is a major risk factor in the development of metabolic syndrome and type 2 diabetes [1, 2]. Diabetes is reaching epidemic proportions with nearly 8.3% of global population diagnosed as diabetic [3, 4]. Acute stress responses mobilize body’s energy reserves to cope with the changed energy requirements in the short-term [5]; however, long-term stress is detrimental to health. Stress responses are coordinated by the corticotropin-releasing factor (CRF) family that comprises four known peptide hormones—CRF and three urocortins (UCN 1–3)—and two G protein-coupled receptors (GPCRs)—CRF_1_ and CRF_2_ [6]. CRF_2_ is encoded by the gene Crhr2 present on chromosome 7 in humans and 6 in mice. CRF is intimately involved in regulation of behavioral and endocrine stress responses by activating the HPA axis via CRF_1_. Urocortins dampen stress sensitivity via activation of the CRF_2_. CRF and UCNs are also involved in regulating cardiovascular function [7, 8] and inflammatory responses [9–12]. Both UCN2 and UCN3 are known to regulate glucose homeostasis in animal models of type 2 diabetes [13, 14]*.* CRF_2_ is the main receptor that mediates stress-coping actions of psychological stressors and downregulation of its expression and function is observed during chronic stress [15].

UCN3 is thought to be a marker for β-cell maturity and function [14]. UCN3 is stored and released with insulin, participating in a negative feedback loop that promotes somatostatin release to ensure timely reduction of insulin secretion [14]. Lack of UCN3 has been linked to excessive insulin release, contributing to the pathophysiology of diabetes [14]. A single intravenous injection of adeno-associated virus encoding urocortin2 (AAV.UCn2) normalized blood glucose in db/db and C57BL/6 mice rendered diabetic with HFD [13]. CRF_2_ is required to mediate therapeutic effects of UCN2, and null mice for CRF_2_ (Crhr2^−/−^) are unable to improve glucose mobilization even in presence of AAV.UCn2 [13]. Thus, CRF_2_ is crucial in mediating effects of UCN2 and probably UCN3 in glucose homeostasis.

Nutritional excess as seen with intake of calorie-rich foods, contributes to the development of type 2 diabetes and metabolic syndrome. Metabolic syndrome refers to a cluster of abnormalities including impaired glucose metabolism, insulin sensitivity, obesity, and dyslipidemia [16–18]. The abnormalities associated with metabolic syndrome are risk factors for cardiovascular diseases and diabetes. In experimental animal models, excess dietary fat consumption contributes to impaired glucose metabolism, leading to development of insulin resistance and hyperglycemia [19]. High-fat diet feeding has been used as a model to induce type 2 diabetes, obesity, and associated phenotypes in mice [20]. Psychological stressors downregulate expression of CRF_2_ receptors [21], and CRF_2_ receptor null mice (Crhr2^−/−^) are more anxious than wild-type littermates [22, 23]. Psychological stressors also drive sex-specific metabolic disturbances in human population studies [1], but whether downregulation of CRF_2_ receptors also drives metabolic disturbances, such as hyperglycemia, insulin resistance, dyslipidemia, and fatty liver associated with nutritional stress, has not been shown. In this study, we asked if impairment of the CRF_2_ receptor contributed to the development of sex-specific metabolic phenotypes during nutritional stress. To test this hypothesis, we subjected Crhr2^−/−^ and wild-type mice of both sexes to 8 weeks of high-fat diet nutritional stress and evaluated metabolic outcomes.

Here, we report that Crhr2 null male, but not female mice develop impaired glucose tolerance on standard chow. In Crhr2 null male mice, fat redistribution and dyslipidemia contributed to the development of metabolic phenotype and insulin resistance. High-fat diet worsened insulin sensitivity in Crhr2 null male mice alone. Sex, diet, and genotype were all significant variables in both glucose tolerance and insulin sensitivity, suggesting that dysregulation of CRF_2_ receptor, a key mediator of stress responses, is involved in the development of diabetes and metabolic syndrome.

## Materials and methods

### Animal studies

All animal procedures were approved by the Institutional Animal Care and Use Committee (IACUC) at the University of California San Francisco and were conducted in accordance with the National Institutes of Health *Guide for the Care and Use of Laboratory Animals.* Generation of Crhr2^−/−^ (C57BL/6 background) mice has been described previously [23]. Crhr2^+/−^ x Crhr2^+/−^ (heterozygous) mice were bred to obtain wild-type (WT), heterozygous (Crhr2^+/−^), and knockout (Crhr2^−/−^) male and female mice as described previously [24]. Littermates of 8–9 weeks of age weighing 18–20 g (female mice) and 25–29 g (male mice) were used in all the studies described. The mice were housed in a room that was temperature (22–25 °C) and light controlled (12-h: 12-h light/dark cycle starting at 7 AM). The mice were fed with a standard chow diet consisting of 9% fat (PicoLab mouse Diet 20 #5058, Lab Supply, Fort Worth, Texas, USA) or high-fat diet (HFD) with 60% kcal fat (Catalog# 12492 Research Diets, NJ USA) for 8 weeks. Mice were group housed with 4–5 mice per cage and provided with enrichment. The entire experiment was repeated once (*n* = 4–5/group/sex) with a total of *n* = 8–10/group/sex. At the end of the experiments, mice were euthanized and blood was collected in heparinized 1-ml syringe from the inferior vena cava. Various tissues were collected for analyses.

### Body mass/weight

Mice were weighed twice weekly throughout the duration of the study. Mice were handled consistently by the same researchers (S.P and S-I.H) to minimize handling-related stress. Change in body mass gain per week was calculated by subtracting the baseline value obtained at the start of the study. Percent (%) change in body mass for chow- or HFD-fed mice was calculated using the formula: final weight in g − initial weight in g/initial weight in g × 100 to allow for comparisons between all groups.

### Food intake

Food intake per cage was calculated by dividing the total food intake per cage/week by number of mice/cage and is considered as a good measure of food intake [25]. Since body weight gain showed sexual dimorphism, food intake per mouse was calculated by dividing food intake per cage/average body weight of the mice per cage (g/g bw). Percent (%) difference in food intake was calculated using the formula: |V1 − V2|(V1 + V2)2 × 100 to allow for comparisons between all groups.

### Glucose tolerance test (GTT)

WT, Crhr2 heterozygous, and Crhr2 null mice of both sexes were fed with standard chow or HFD for 8 weeks. Intraperitoneal glucose tolerance test (IP-GTT) was conducted to assess the disposal of glucose by measuring glucose level after fasting for 14–16 h. The testing was performed at a fixed time to avoid circadian variations in blood glucose levels. Per day, maximum of two cages of either male or female sex (total of 8–10 mice from two cages) were used to perform GTT or insulin tolerance test. Mice were weighed after fasting, and baseline (pre-injection or time 0 min) level of glucose was measured. Mice were injected ip with 2 g/kg glucose (Sigma, St. Louis, MO, USA), and glucose levels were measured at 30, 60, and 120 min. Blood samples were obtained by tail nick, and glucose levels were monitored using the Alpha Trak2 glucose monitor and strips (Abbotts, USA).

### Insulin tolerance test (ITT)

WT, Crhr2 heterozygous, and Crhr2 null mice of both sexes were fasted for 6 h and body weight was recorded. Blood sample was collected by tail nick and baseline (0 min) glucose was measured using the Alpha Trak2 glucose monitor and strips (Abbotts, USA). Mice were injected ip with 0.375 U/kg human insulin (Novolin, Novo Nordisk), and glucose levels were measured at 15, 30, 45, and 60 min after insulin injection.

### Insulin assay and plasma lipid profile analysis

Blood was collected in heparinized vials for insulin assay and plasma lipid profile analysis. Plasma insulin was assayed using kit from Mesoscale diagnostics (www.mesoscale.com, USA). Cholesterol, triglycerides, and high-density lipoprotein (HDL) were measured using the Siemens Advia chemistry analyzer (Siemens Healthcare Diagnostics Inc., USA). LDL was calculated using the formula: LDL Cholesterol = Total cholesterol − (HDL Cholesterol + Triglycerides/5).

### Histology

Tissues were collected and fixed in 4% paraformaldehyde for 14–16 h. Tissues were paraffin embedded, and 4–6 μm sections were cut and stained with hematoxylin-eosin at the Mouse Histology Core Facility, University of California San Francisco. Livers from four to five mice/group were imaged, and six to eight fields/liver were captured. A liver pathologist (Dr. Aras Mattis, MD, PhD), blind to the treatment groups, helped with analysis of liver sections.

### Statistical analysis

We considered genotype, diet, and sex as independent variables. GTT and ITT data was analyzed using repeated-measure analysis of variance (ANOVA) followed by Sidak’s post hoc analysis. For all other outcomes, significant main effects of each variable and their interactions were determined using three-way ANOVA. When significant main effects were present, Tukey’s multiple comparisons were performed. When comparing two groups, Student’s *t* test was used. A *p* value < 0.05 was considered significant. All statistical analyses were conducted using the GraphPad Prism 7.0™ software.

## Results

### Crhr2-deficient male mice gained more body mass than Crhr2^−/−^ female mice

**Fig. 1:**
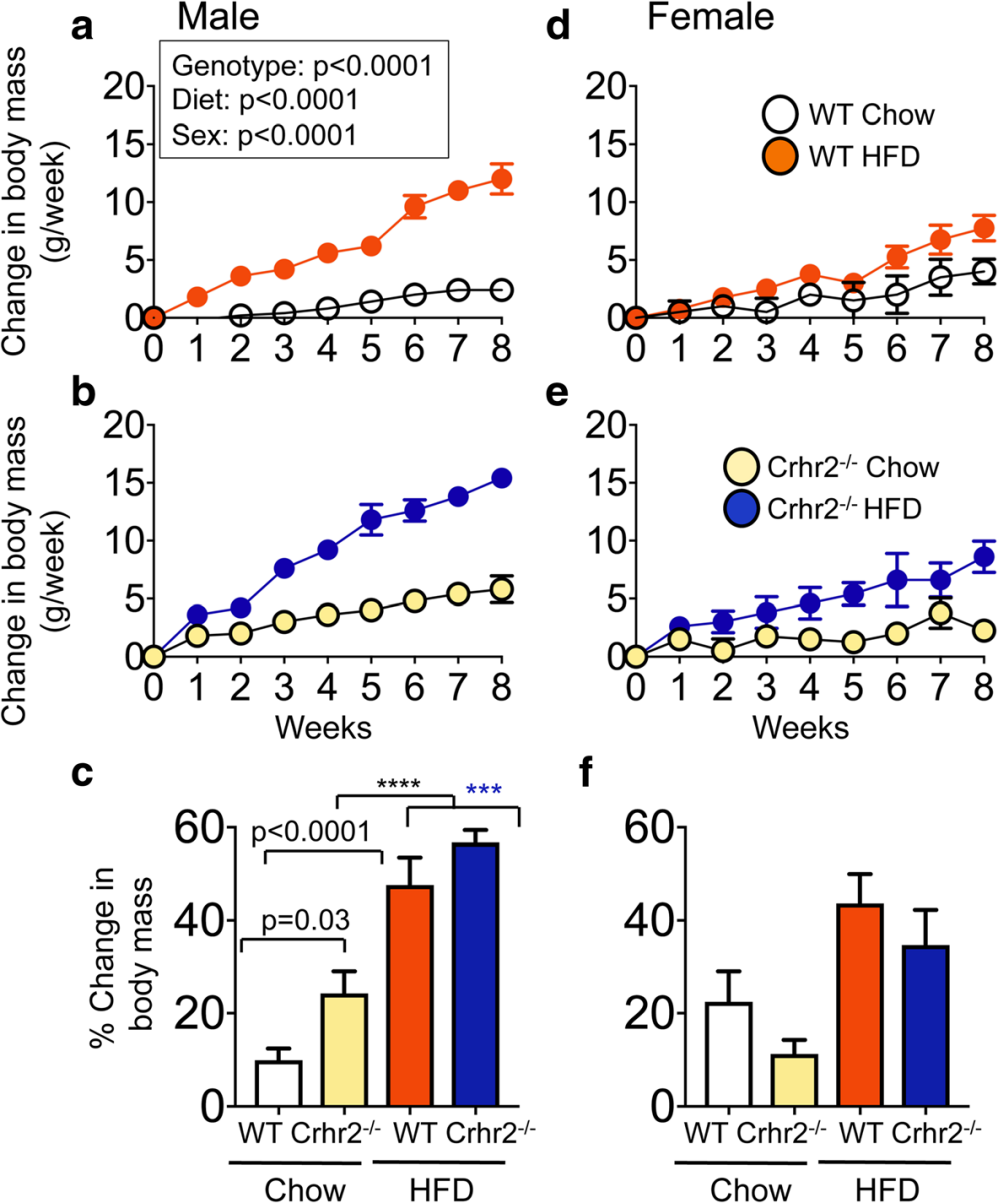
Crhr2^−/−^ male mice gain more body mass. Line graphs showing weekly change in body mass in **a**, **b** male (*n* = 9/group) and **d**, **e** female (*n* = 9/group) WT and Crhr2 null mice on chow and HFD. Data is presented as change in body mass per week compared to baseline. **c**, **f** Bar graphs showing percent (%) change in body mass for chow- or HFD-fed mice. Three-way ANOVA and post hoc Tukey’s multiple comparisons. ****p < 0.0001, Crhr2^−/−^ male mice chow vs. HFD and ***p = 0.0005, WT male HFD vs. Crhr2^−/−^ male HFD

Body mass gain is associated with metabolic syndrome and type 2 diabetes. We determined the contribution of CRF_2_ receptor (genotype) and sex on body mass gain and food intake. Genotype, sex, and diet were significant independent variables for body weight mass gain and also showed significant interactions (Fig. 1 and Additional file 1). At the end of 8 weeks, WT male mice gained ~ 10% body mass on chow and ~ 47% on HFD (Fig. 1a, c). Crhr2^−/−^ male mice gained ~ 21% body mass on chow and ~ 53% on HFD (Fig. 1b, c). WT female mice gained ~ 20% body mass on chow and ~ 43.6% on HFD (Fig. 1d, f). Crhr2^−/−^ female mice gained only ~ 10% body mass on chow ~ 41% on HFD (Fig. 1e, f), and the body mass gain between chow and HFD in WT and Crhr2^−/−^ female mice was not statistically significant. Crhr2^+/−^ male and female mice also showed significant body mass gain as compared with their chow-fed controls (Additional file 1).

### Crhr2-deficient male mice increased chow intake, whereas Crhr2-deficient female mice decreased HFD intake

**Fig. 2:**
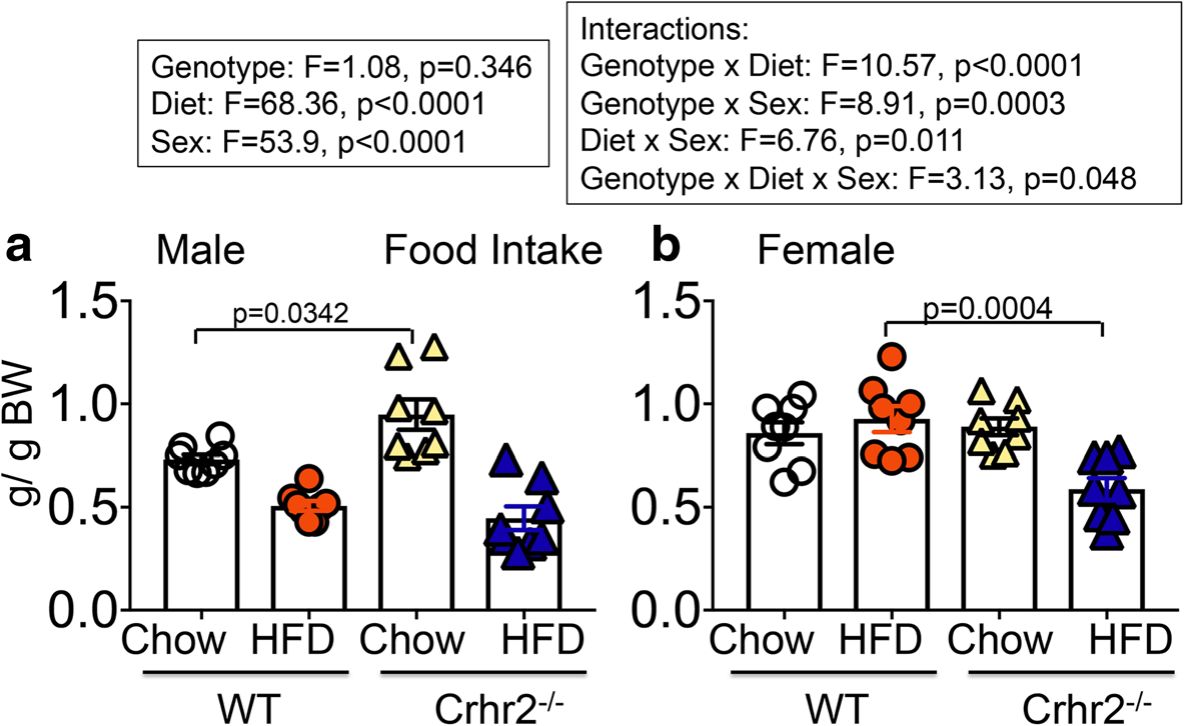
Crhr2^−/−^ male mice increase chow diet intake. Column bar graphs showing weekly average food intake per mouse in g/g body weight. **a** Crhr2 null male mice consumed 31.68% more chow per week than WT littermates (*p* = 0.0342, *n* = 8/group) (**b**) WT female mice consumed 27.57% more HFD per week than Crhr2 null littermates (*p* = 0.0004, *n* = 8/group). Three-way ANOVA and post hoc Tukey’s multiple comparisons

Genotype, sex, and diet showed significant interaction for food intake (Fig. 2). Male Crhr2^−/−^ mice increased chow intake by 25.71 and 36.89% compared with WT and Crhr2^+/−^ mice (*p* = 0.0342 and *p* = 0.0013 respectively, Fig. 2a and Additional file 2). No significant difference in HFD intake in male mice was noted (Fig. 2a). In contrast to male mice, chow intake in female of different genotypes did not differ, whereas female Crhr2^−/−^ consumed 44.67% less HFD compared with WT female mice (*p* = 0.0004, Fig. 2b).

### Crhr2 genotype affected blood glucose levels in a sexually dimorphic manner

**Fig. 3:**
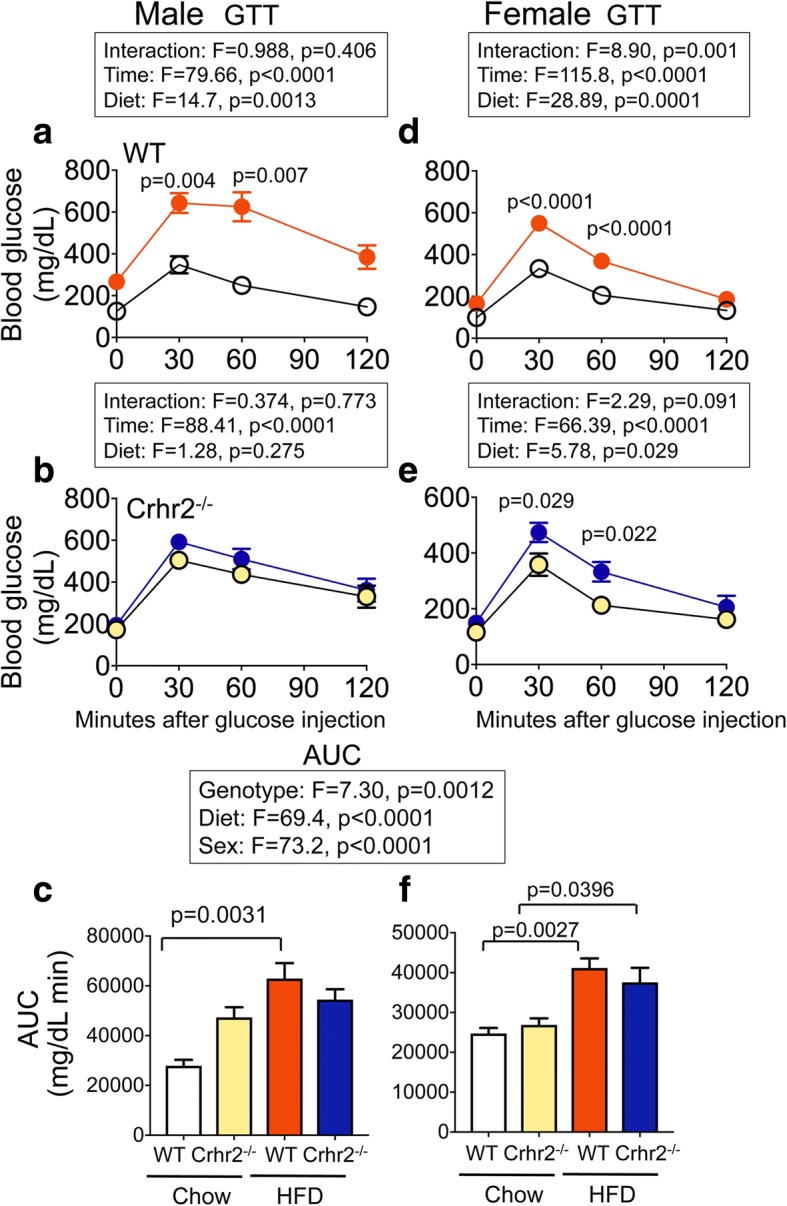
Crhr2^−/−^ male mice show elevated blood glucose levels on chow. In GTT, blood glucose was measured by tail vein sampling before glucose administration (baseline; 0 min) and at 30, 60, and 120 min after a bolus of intraperitoneal glucose (2 g/kg) injection. Repeated-measure ANOVA followed by Sidak’s post hoc test was used to analyze GTT data. **a** In WT male mice, blood glucose levels were significantly elevated at 30 and 60 min time points on HFD vs. chow. **b** In Crhr2^+/−^ male mice, blood glucose levels did not differ between the diets, but significantly changed over time. **c** Glucose clearance rate, as reflected by AUC, was significantly elevated in HFD- vs. chow-fed WT male mice, whereas AUC in Crhr2^−/−^ male mice was comparable on HFD and chow. In female (**d**) WT and (**e**) Crhr2^−/−^ HFD-fed mice, significant increases in blood glucose levels were noted at 30 and 60 min post glucose injection. **f** AUC was higher in HFD-fed vs. chow-fed female mice. (*n* = 8/group/sex)

To determine the contribution of CRF_2_ receptor and sex in glucose clearance, we performed GTT and ITT at the end of 8 weeks. Genotype, diet, and sex were all variables in regulation of glucose clearance (Fig. 3). As expected, HFD-fed WT male mice showed an increase in the area under the curve (AUC) compared with chow-fed controls (Fig. 3a, c). Surprisingly, chow-fed Crhr2^−/−^ male mice had AUC that was equivalent to that of HFD-fed Crhr2^−/−^ male mice (Fig. 3b, c). On the other hand, HFD-fed female WT and Crhr2^−/−^ mice showed impaired IP-GTT responses with significantly elevated blood glucose levels and AUC compared to chow-fed controls (Fig. 3d–f). HFD-fed Crhr2^+/−^ (heterozygous) male and female mice also showed significant increases in blood glucose levels and AUC compared to chow-fed controls (Additional file 3).

### Crhr2-deficient male mice exhibited impaired insulin sensitivity, whereas female mice were protected

**Fig. 4:**
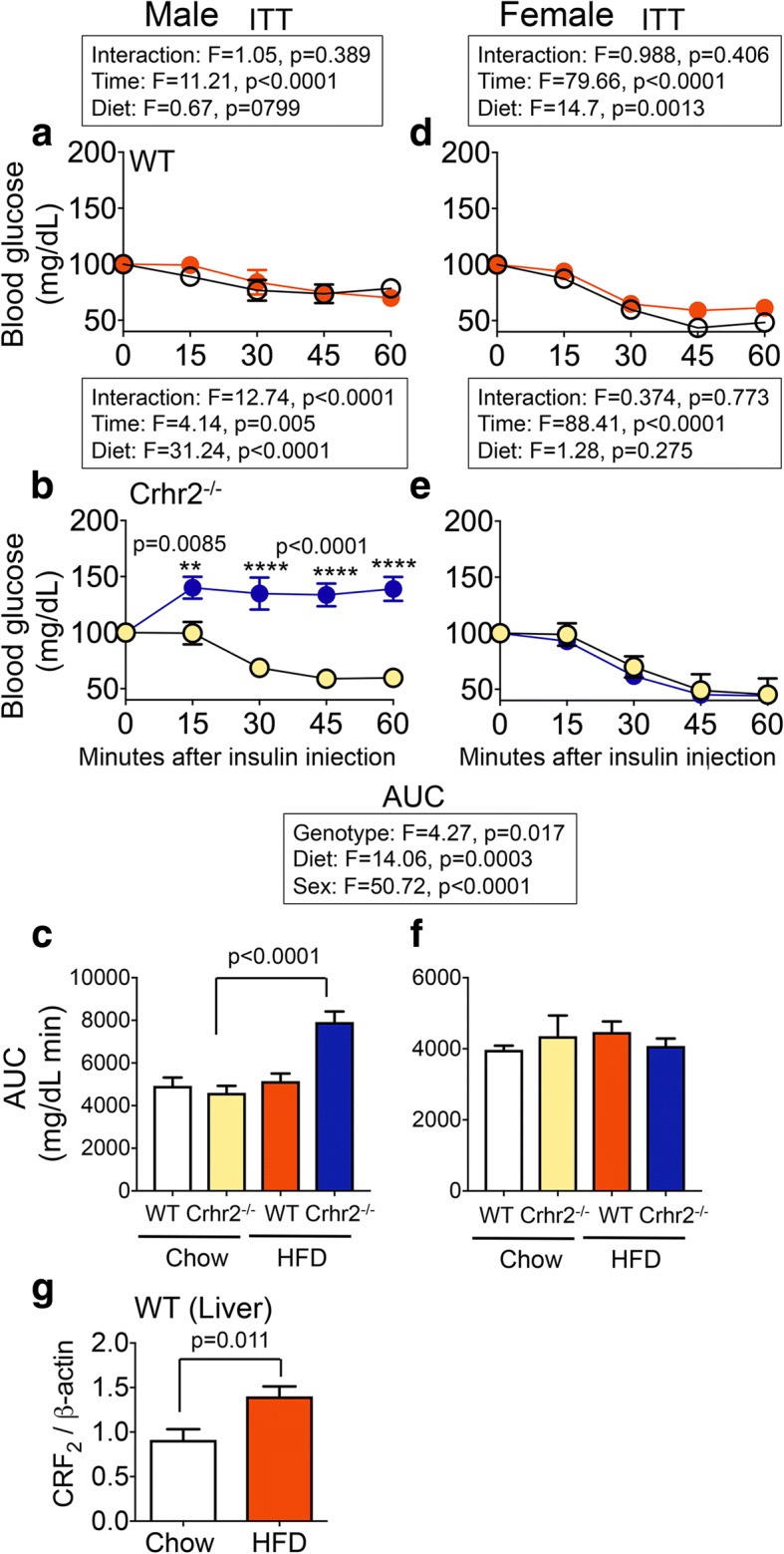
Crhr2^−/−^ male mice have impaired blood glucose clearance in response to insulin challenge. In ITT, blood glucose levels were measured by tail-vein sampling before (baseline; 0 min) and at 15, 30, 45, and 60 min after ip insulin administration in chow- and HFD-fed mice. Repeated-measure ANOVA followed by Sidak’s post hoc test was used to analyze ITT data. **a**–**c** Male WT mice showed normal response to ITT challenge. HFD-fed Crhr2^−/−^ male mice had higher blood glucose levels at all times examined and a 2-fold higher AUC. **d**–**f** Female WT and Crhr2^−/−^ mice retained normal ITT responses on chow and HFD. *n* = 8/group/sex. **g** CRF_2_ receptor levels were ~ 1.5-fold higher in livers of WT male on HFD vs. chow. Expression levels were normalized to β-actin, a housekeeping gene. Student’s *t* test, *n* = 8/group

Genotype, diet, and sex were all variables in regulation of insulin sensitivity as evidenced by impaired glucose clearance after a bolus of insulin injection in ITT (Fig. 4a–e). WT male mice on chow and HFD similarly decreased blood glucose levels in ITT (Fig. 4a–c). HFD-fed Crhr2^−/−^ male mice had significantly elevated glucose levels compared to chow-fed Crhr2^−/−^ mice at all time points (Fig. 4b). The AUC was nearly twofold higher in HFD-fed vs. chow-fed Crhr2^−/−^ male mice (*p* < 0.0001, Fig. 4c). Female Crhr2^−/−^ mice and Crhr2^+/−^ mice of both sexes retained normal glucose responses in ITT (Fig. 4d–f and Additional file 4). Since liver is a major site of insulin action that regulates glucose release, next, we examined whether HFD modulated CRF_2_ receptor expression in the livers of WT male mice. CRF_2_ receptor expression increased by ~ 1.5-fold in the livers of HFD-fed mice compared to chow-fed mice (Fig. 4g). Taken together, these data highlight the role of stress receptor, CRF_2_ in mediating sexually dimorphic peripheral insulin resistance.

### Male, but not female mice on HFD have significantly higher plasma insulin levels

**Fig. 5:**
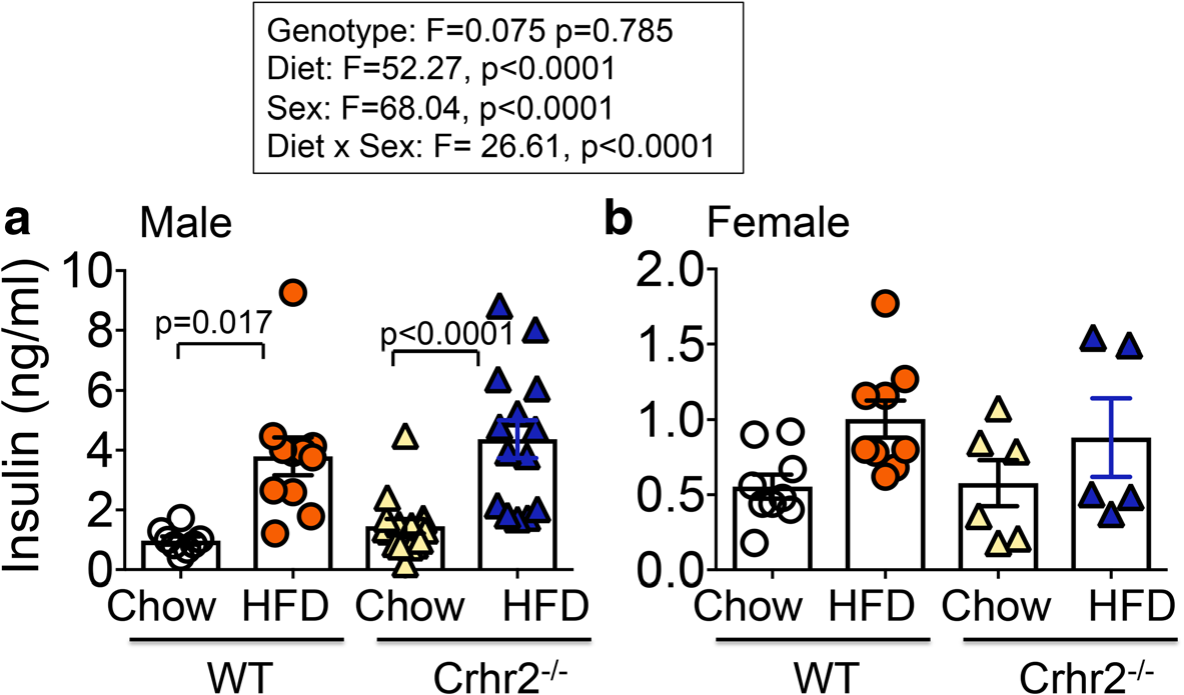
Male mice have increased plasma insulin levels on HFD. Column graphs showing plasma insulin levels in chow- and HFD-fed male and female mice. **a** Significant increases in insulin levels were seen in male WT and Crhr2^−/−^ mice (HFD vs. chow, n = 9/group). **b** HFD intake did not significantly increase plasma insulin levels in female WT and Crhr2^−/−^ mice (*n* = 9/group). Three-way ANOVA and post hoc Tukey’s multiple comparisons

As Crhr2 null mice were insulin resistant, we next assessed changes in plasma insulin levels in mice of both sexes in response to HFD consumption. Genotype, sex, and diet were significant independent variables and also showed significant interaction (Fig. 5). Plasma insulin levels were significantly elevated in HFD-fed WT, Crhr2^−/−^, and Crhr2^+/−^ male mice compared to their respective chow-fed controls (Fig. 5a and Additional file 5). HFD intake did not significantly alter plasma insulin levels in female mice of any genotype compared to chow-fed control mice (Fig. 5b and Additional file 5).

### Sex and diet were variables in fat mass gain and/or redistribution

**Fig. 6:**
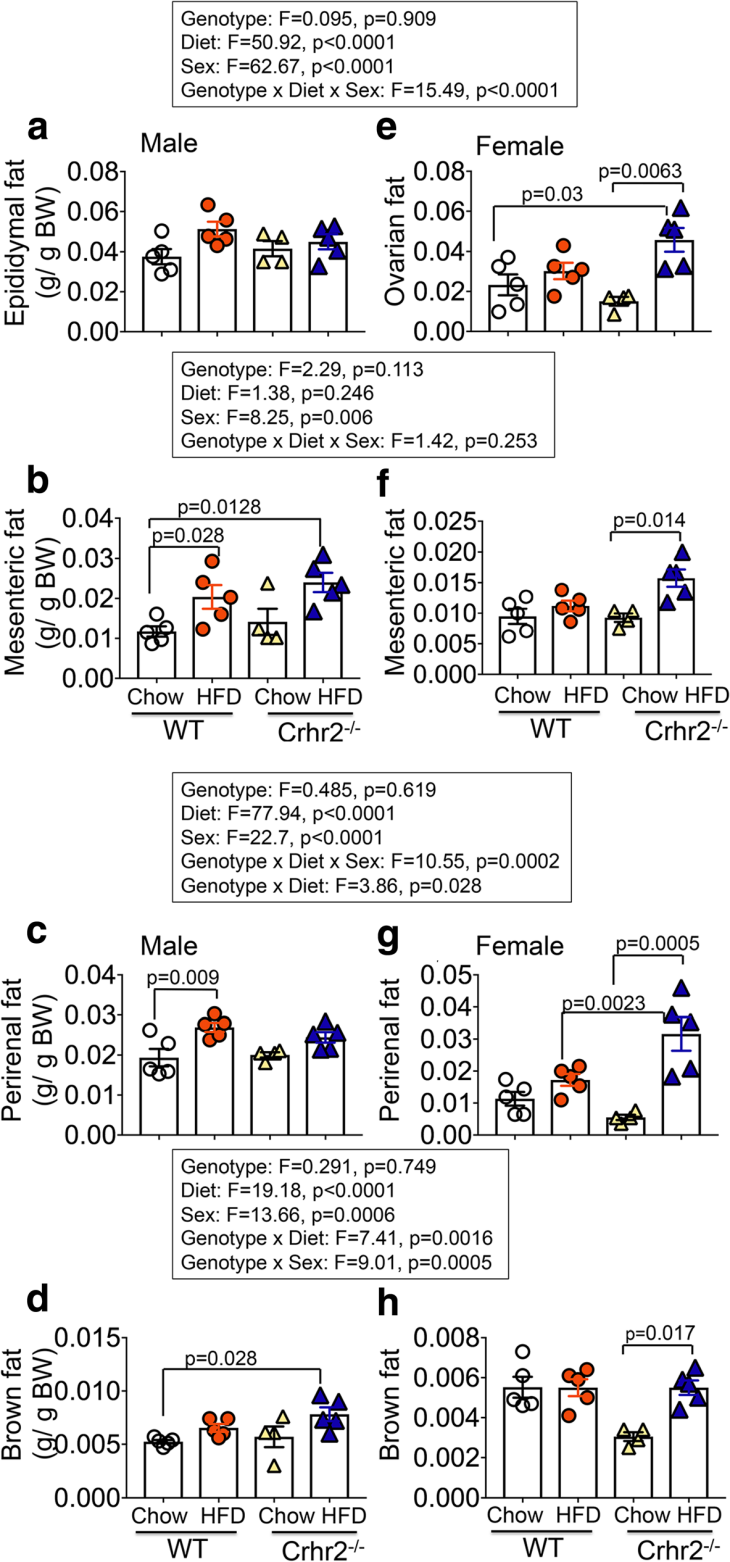
Sex-specific fat mass gain and/or redistribution on chow vs. HFD. Column bar graphs showing fat mass gain over 8 weeks on chow vs. HFD consumption in male and female mice. Four types of fat mass—gonadal (epididymal/ovarian), mesenteric, perirenal, and brown—were assessed. **a** Diet did not change gonadal fat depots in male mice. **b** HFD-fed WT and Crhr2^−/−^ male mice increased their mesenteric fat depots by 53.79% and 145.75%, respectively compared with WT chow-fed controls. **c** HFD-fed WT male mice increased their perirenal fat depots by 32.67% vs. chow. **d** HFD-fed male Crhr2^−/−^ mice increased their brown fat depots by 253.1% compared with chow-fed WT mice. **e** HFD-fed female Crhr2^−/−^ mice gained 103.91% more ovarian fat vs. chow diet. **f** HFD-fed female Crhr2^−/−^ mice increased their mesenteric fat depots by 38% vs. chow. **g** HFD-fed female Crhr2^−/−^ mice increased their perirenal fat mass by 140% vs. chow diet and by ~ 59% compared with HFD-fed WT female mice. **h** HFD-fed female Crhr2^−/−^ mice increased their brown fat depots by 60.86% vs. chow controls. *n* = 5/group/sex. Three-way ANOVA and post hoc Tukey’s multiple comparisons

Visceral fat mass gain and/or fat mass redistribution are factors that contribute to metabolic disorder and peripheral insulin resistance. Changes in four different fat types—gonadal (epididymal or ovarian), mesenteric, perirenal, and brown fats—were examined. Sex and diet were variables and showed interaction for fat mass gain (Fig. 6a–h). In male mice, HFD intake did not significantly change gonadal (epididymal) or brown fat mass in WT and Crhr2^−/−^ mice compared to their respective chow-fed controls (Fig. 6a, d). There were significant increases in mesenteric and perirenal fat mass in HFD-fed WT male mice compared to chow-fed controls (Fig. 6b, c). Significant differences in epididymal and perirenal fats were observed between chow- and HFD-fed Crhr2^+/−^ male mice (Additional file 6). In female mice, HFD intake in Crhr2^−/−^ mice resulted in significant fat mass gained in all four types of fats examined compared with chow controls (Fig. 6e–h).

### Crhr2-deficient male mice showed dyslipidemia, but female mice were protected

**Fig. 7:**
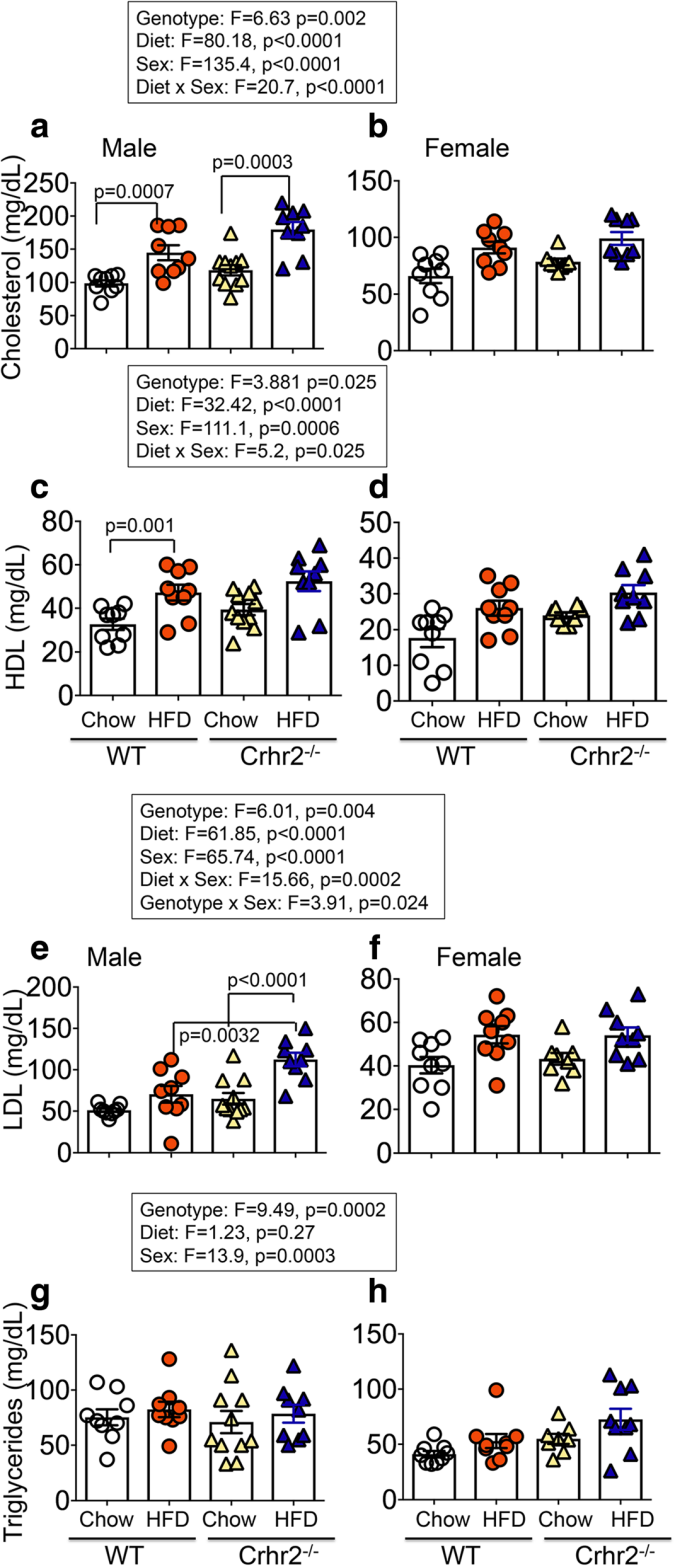
Male mice have increased plasma lipid levels. Column bar graphs show plasma lipid profiles in male and female mice. Plasma cholesterol, HDL, triglycerides, and LDL levels were determined. **a** HFD-fed male WT and Crhr2^−/−^ mice had 37.7%, and 40.9% higher blood cholesterol levels vs. chow diet, respectively. **b** In female WT and Crhr2^−/−^ mice, HFD consumption resulted in smaller, non-significant increases in blood cholesterol levels vs. chow. **c** HFD-fed male WT mice had 36.1% higher HDL levels vs. chow. **d** In female mice, HDL levels did not differ between HFD vs. chow. **e** HFD-fed male Crhr2^−/−^ mice had ~ 53.0% higher calculated LDL levels vs. chow-fed Crhr2^−/−^ and HFD-fed WT mice. **f** In female mice, diet did not change LDL levels. **g**, **h** In male and female mice, diet did not change triglycerides levels. *n* = 8/group/sex. Three-way ANOVA and post hoc Tukey’s multiple comparisons

Changes in two or more plasma lipid levels (dyslipidemia) are associated with diagnosis of metabolic syndrome and type 2 diabetes. Plasma lipid panel that included cholesterol, high-density lipoprotein (HDL), and triglycerides was determined using mass spectrometry. Low-density lipoprotein (LDL) levels were calculated as described in methods. Genotype, sex, and diet were significant independent variables in plasma lipid levels and also showed significant interaction (Fig. 7).

### Cholesterol

In male mice, HFD intake resulted in elevated plasma cholesterol levels by 37.7% in WT, 50.3% in Crhr2^+/−^, and by 40.9% in Crhr2^−/−^ compared with chow-fed controls (Fig. 7a and Additional file 7a). In female mice, HFD intake did not result in significant increases in plasma cholesterol compared with chow fed controls (Fig. 7b and Additional file 7b).

### HDL

In male mice, plasma HDL levels increased by 36.1% in WT and by 38.3% in Crhr2^+/−^ compared with chow-fed controls (Fig. 7c and Additional file 7c). In female mice of all three genotypes, plasma HDL levels showed did not differ between chow and HFD (Fig. 7d and Additional file 7d).

### LDL

In male mice, plasma LDL levels increased by 53.6% in Crhr2^−/−^ and by 68.0% in Crhr2^+/−^ compared with chow-fed controls (Fig. 7e and Additional file 7e). Importantly, HFD-fed Crhr2^−/−^ male mice had approximately twofold higher LDL levels compared with HFD-fed WT mice (Fig. 7e). In female mice, LDL levels did not differ between HFD- and chow-fed mice (Fig. 7f and Additional file 7f).

### Triglycerides

In male mice of either genotype, triglyceride levels did not differ between HFD vs. chow (Fig. 7g). In female WT and Crhr2^−/−^ mice, triglyceride levels did not differ between HFD vs. chow (Fig. 7h). Interestingly, triglyceride levels in chow-fed Crhr2^+/−^ female mice were significantly higher compared with chow-fed WT and Crhr2^−/−^ (Additional file 7 h).

### Crhr2-deficient male mice develop microvesicular liver steatosis

**Fig. 8:**
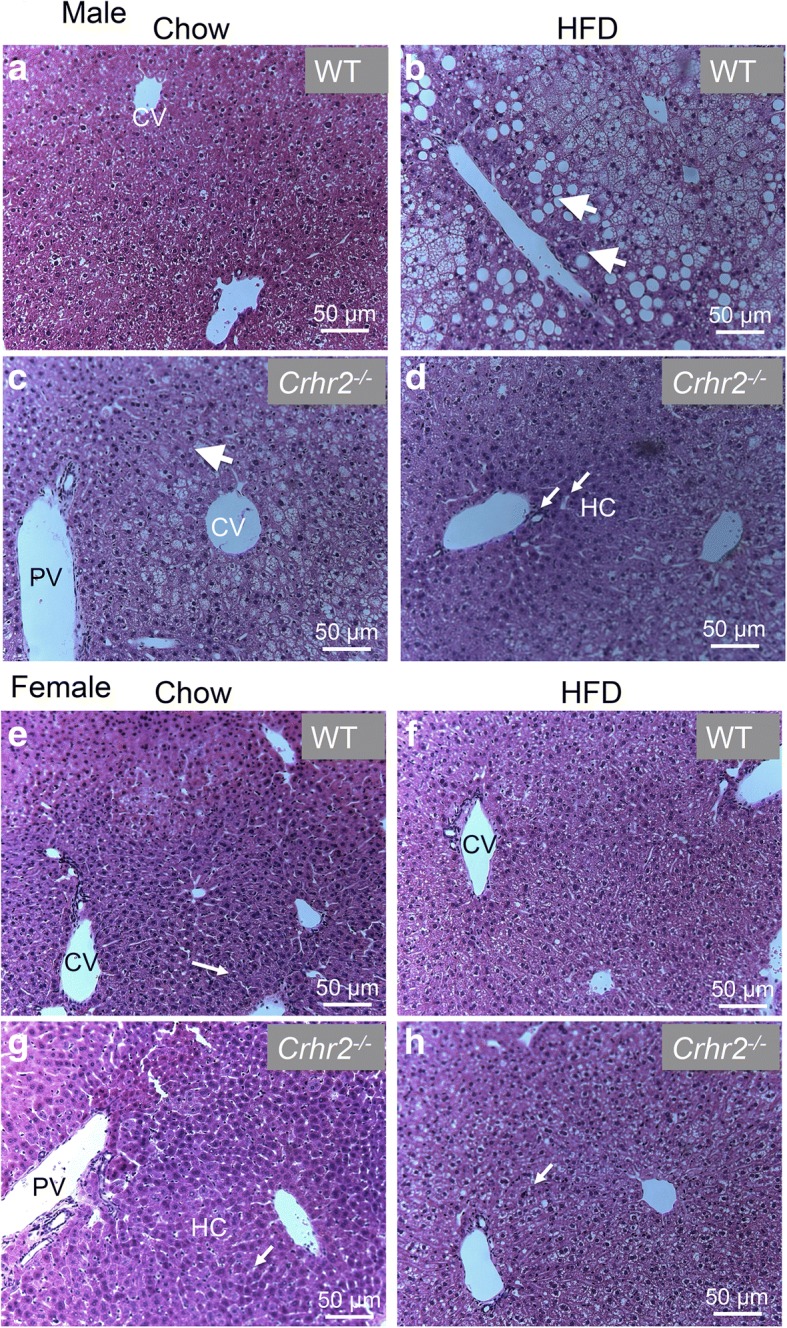
Male mice show hepatic steatosis. Representative micrographs showing H&E stained liver sections from WT and Crhr2^−/−^ mice of both sexes. **a** Normal hepatocyte morphology is evident in chow-fed WT male mice. **b** Hepatocytes show ballooning with swollen sinusoids (arrows) and fat droplets (arrowheads) suggesting presence of macrovesicular steatosis in HFD-fed WT male mice. **c**, **d** Hepatocytes show ballooning and microvesicular steatosis in both chow- and HFD-fed Crhr2 null male mice. **e**, **h** The livers of WT and Crhr2 null female mice on either diet show normal hepatocyte morphology and no evidence of steatosis. Scale bars = 50 μm. CV and PV: central and portal veins, respectively; HC: hepatic cords

Lipid accumulation in the liver is a characteristic of a myriad of diseases such as type 2 diabetes and metabolic syndrome. H&E-stained sections revealed macrovesicular steatosis in livers of HFD-fed WT male compared with chow-fed controls with fat droplets vacuoles evident (Fig. 8 a, b, arrows). Surprisingly, livers of chow-fed Crhr2^−/−^ male mice exhibited microvesicular steatosis with swollen sinusoids evident (Fig. 8c, arrows) and HFD consumption for 8 weeks did not further worsen liver steatosis in Crhr2^−/−^ male mice (Fig. 8d). In sharp contrast to male mice, chow- or HFD-fed female WT or Crhr2^−/−^ mice did not show any signs of steatosis (Fig. 8e, f).

## Discussion

Chronic stress is known as a factor contributing to the development of metabolic syndrome, type 2 diabetes, and many other pathophysiologies. In this study, we report several novel observations; mice lacking the stress receptor, CRF_2_, show impaired glucose clearance and microvesicular liver steatosis on standard chow diet in a sexually dimorphic manner. Short-term nutritional stress rendered male mice of Crhr2-deficient genotype insulin resistant and dyslipidemic, whereas female mice of Crhr2 genotype were protected, in the time period examined. CRF_2_ receptor activation and function is required for feedback to the HPA axis to bring systems back to homeostasis after stress responses. CRF_2_ receptor dysregulation is reported in chronic stress [21, 26]. Prospective cohort studies have shown that perceived stress increases risk of developing type 2 diabetes in men, but not in women by approximately twofold and a 1.4 higher odds ratio [1]. Our findings that only male mice with Crhr2 genotype, but not female mice develop impaired glucose clearance and other metabolic phenotypes on short-term nutritional stress, is in agreement with clinical reports that men are more prone to developing type 2 diabetes under stressful conditions. Haploinsufficiency of CRF_2_ may be akin to downregulation of CRF_2_ receptor under chronic stress conditions and increasing the risk of developing metabolic syndrome and type 2 diabetes.

Male and female mice gained body mass differently on a fat-rich diet. In animal models, male C57BL/6 mice gain significantly more body and fat mass than female mice on HFD [26]. We found that lack or haploinsufficiency of stress receptor, CRF_2_, rendered male mice even more prone to body and fat mass gain on a fat-rich diet than WT male mice, whereas female mice of Crhr2 genotype were less susceptible to fat-rich diet-induced changes, at least in this short-term nutritional stress model. Since body mass gain was significantly different between the genotypes and sexes, fat mass and food intake were normalized to body mass. On a standard diet, Crhr2 null male mice gained ~ 12% more body mass than WT littermates. The differences in body mass gain between WT and Crhr2 null male mice could be accounted for by increased chow diet intake per week over the 8-week period. On a fat-rich diet, Crhr2 null male mice gained ~ 6% more body mass than WT littermates, but HFD consumption did not differ between the two genotypes. Female mice of either genotype showed increase in body weight on HFD, but the body mass gains were not significantly different from that gained on chow, suggesting that female mice may handle short-term nutritional stress overload better than male mice.

We found genotype and sex as significant modifiers of the effect of diet for several metabolic outcomes. First, only male mice of Crhr2 genotype had worse glucose clearance on standard chow diet. A recent systematic study that characterized effects of sex and body weight on metabolic outcomes on a high-fat diet in C57BL/6 mice found that male mice show worse glucose clearance on high-fat diet than female mice [26]. Furthermore, this published study used nearly 300 mice per sex to characterize metabolic phenotype on breeder’s chow and HFD to create a reference database [26]. Our sexually dimorphic metabolic phenotypes in C57BL/6 mice are in agreement with this reference data bank.

Genotype, diet, and sex also were significant modifiers of peripheral insulin responses. In insulin tolerance studies, only male Crhr2 null mice were hyperglycemic on a fat-rich diet. Blood glucose level remained significantly elevated in HFD-fed Crhr2 null males after insulin injection and throughout the duration of the experiment (60 min), whereas glucose levels returned to baseline in HFD-fed WT male and female mice. We used the lowest possible insulin dose, as female Crhr2^−/−^ mice did not tolerate higher doses of insulin, becoming severely hypoglycemic and had to be given a bolus of glucose. At the end of 8 weeks of fat-rich diet consumption, CRF_2_ receptor levels increased in livers of WT mice suggesting diet-induced compensation of the stress axis. Others have shown that palatable food intake feeds back to the HPA axis to better cope with stress responses [27, 28]. A combination of low dose of insulin used in ITT, increase in CRF_2_ receptor levels, and possible association of CRF_2_ receptor with ancillary proteins [29] that may differ between the sexes, may explain this sexual dimorphism in metabolic responses to nutritional stress. It might be possible that CRF_2_ receptor levels show bimodal responses to nutritional stress, with levels increasing during short-term, but decreasing with long-term (> 8 weeks) HFD consumption. Consistent with this notion, bimodal levels of CRF_2_ receptors have been shown in response to acute vs. chronic inflammatory stress in other experimental animal models [9].

Enhanced release of glucagon, glycogenolysis, and peripheral insulin resistance may explain hyperglycemia in Crhr2 null mice. Chronic insulin resistance leads to hyperinsulinemia and resulting inactivation of insulin signaling pathways [30–32]. In rodent pancreas, paracrine communication between α- and β-cells resulted in insulin-inhibiting glucagon release. Selective inactivation of insulin receptor in pancreatic α-cells is known to upregulate glucagon release [33]. Delivery of anti-insulin antibodies in rat pancreas resulted in increased glucagon release [34]. High blood glucose levels also stimulated glucagon release [35]. Thus, a compromised stress response as in the Crhr2 null mice might worsen metabolic function with higher or dysregulated release of glucagon causing impaired glucose clearance and insulin resistance. The next steps would be to determine insulin and glucagon release from isolated islet cultures from WT and Crhr2^−/−^ mice exposed to different glucose concentrations. The exact mechanism of the insulin-induced hyperglycemia observed in our experimental model is yet to be investigated.

CRF_2_ receptors are expressed on pancreatic δ-cells and may be involved in the release of somatostatin as well as glucagon from δ- and α-cells, respectively. Somatostatin and glucagon in turn are known regulators of insulin release from β-cells [36]. Thus, CRF_2_ receptor dysfunction may regulate insulin release via inhibiting somatostatin and glucagon release in a secondary messenger-dependent manner. We have previously shown that CRF_2_ receptors can alter intracellular concentrations of both cAMP and Ca^2+^ [29, 37]. We have also shown sexual dimorphism in Ca^2+^-dependent signaling in pancreatic acinar cells in murine model of acute stress [24]. Furthermore, we have shown that loss of CRF_2_ receptors downregulated UCN3 expression [12]. Lack of UCN3 in turn might cause excessive insulin release, contributing to the pathophysiology of diabetes [14].

Fat depots contribute to peripheral insulin resistance. Male, but not female mice showed increased plasma insulin levels on a fat-rich diet. HFD-fed male mice significantly increased mesenteric and perirenal fat mass depots, but no changes in gonadal or brown fat mass were observed. HFD-fed WT female mice did not show any significant gain in fat mass, whereas Crhr2 null female mice showed significant increases in gonadal, perirenal, and brown fat depots, despite decreasing their HFD consumption. The increase in gonadal fat mass may in part explain why Crhr2 null female mice take longer to conceive than WT females. This observation is consistent with others who report that temporary nutritional stress causes infertility in female mice [38]. Moreover, increase in brown fat depot may explain why female mice are protected from fat-rich diet-induced metabolic phenotypes in this acute period of nutritional stress. Thus, Crhr2 genotype predisposes males and females differently to body and fat mass gain on a fat-rich diet.

Lipid accumulation in livers is another observed phenotype of metabolic syndrome. Non-alcoholic fatty liver is more prevalent in men than women and diet is a known risk factor. Here, we found that Crhr2 genotype is also a sexually dimorphic risk factor for liver fat accumulation. Male mice with Crhr2 genotype showed microvesicular hepatic steatosis on chow diet as well as on a fatty diet. Insulin resistance has been shown to cause hepatic steatosis [39] although the mechanisms remain to be established [40, 41]. Crhr2 null mice were hyperinsulinemic on a fat-rich diet. Hyperinsulinemia as result of insulin resistance causes hepatic steatosis and deposition of lipid droplets in liver cells due to “selective insulin resistance” [42, 43]. In agreement with other studies [26], we also found that WT C57BL/6 male mice showed macrovesicular steatosis on a fatty diet, whereas female WT and Crhr2 mice did not show any evidence of hepatic lipid accumulation in this acute phase of fatty diet consumption.

Blood cholesterol, HDL, and LDL levels are known to increase on a fatty diet. In rodents, depending upon several factors, blood triglyceride levels may or may not change on a fatty diet [26]. In our study, blood cholesterol and HDL levels increased in Crhr2 null male, WT male, and female mice on HFD compared with chow-fed controls. Crhr2 null male mice also had elevated calculated LDL levels. Female mice of Crhr2 genotype were protected from HFD-induced dyslipidemia.

## Conclusions

Our study is the first to report that downregulation of stress receptors, CRF_2_, drives metabolic disturbances in response to nutritional stress in a sexually dimorphic manner. Stress receptor dysfunction resulted in aberrant glucose clearance, insulin resistance, increased adiposity, dyslipidemia, and fatty liver in Crhr2^−/−^ male mice. Sex was a significant variable in several outcomes analyzed in this study, with female sex being protective for acute nutritional stress that may be explained by reduced fat-rich diet intake and increased brown fat depots.

## Perspectives and significance

**Fig. 9:**
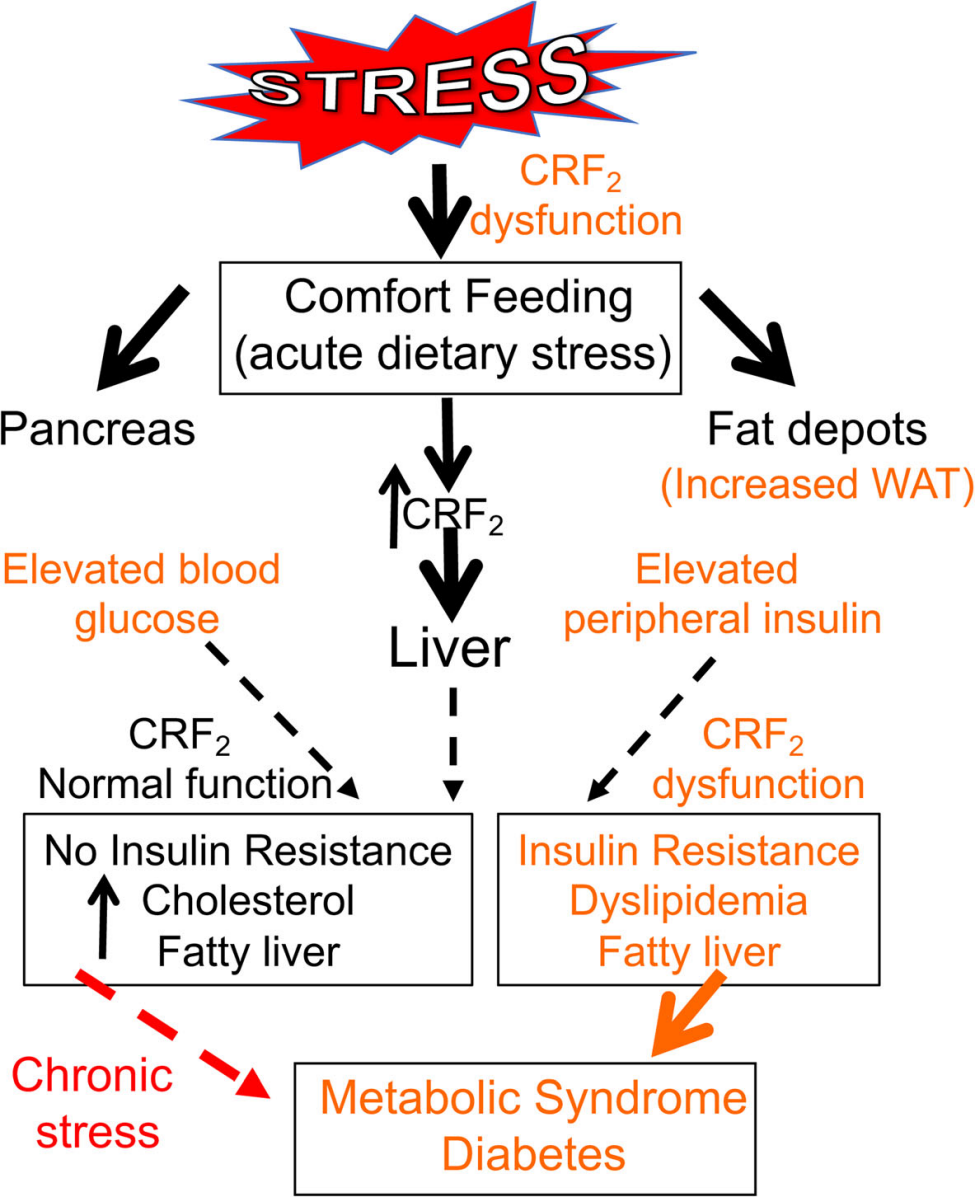
Stress receptor CRF_2_ dysregulation exacerbates metabolic outcomes of acute dietary stress. Acute or short-term fatty food consumption perturbs function in multiple target organs, including liver, pancreas, and fat depots, collectively, these manifested symptoms constitute metabolic syndrome and diabetes. One compensatory mechanism is to increase CRF_2_ receptor expression/function in the liver and other target tissues in order to bring function back to homeostasis. If stressors are removed, function can return to homeostasis (black arrows), but chronic stress exposure may ultimately result in frank metabolic syndrome. Dysregulated CRF_2_ receptor function (as in Crhr2^−/−^ mice) compromises metabolic function even in absence of nutritional stress and puts one on a trajectory to develop metabolic syndrome and diabetes, with males at a greater risk than females. Chronic stress, including consumption of fat- or calorie-rich diets worsens metabolic functions. Thus, CRF_2_ receptor is a sexually dimorphic risk factor for development of metabolic syndrome and associated diseases such as type 2 diabetes

Our studies provide insights into how stress that is often coupled with intake of calorie-rich diet or comfort feeding might contribute to development of metabolic syndrome and type 2 diabetes (Fig. 9). Stress does not target one organ or system, but has pleiotropic effects. CRF_2_ receptor deficiency compromised function in multiple organs. CRF_2_ receptor dysregulation predisposed male mice that are not obese or overweight, to be at greater risk for developing collective symptoms associated with metabolic syndrome and diabetes. Thus, modulation of stress receptor function in a sex-specific manner may help with therapeutic targeting of metabolic syndrome and associated diseases, such as type 2 diabetes.

## Additional files

Additional file 1: Crhr2^+/−^ male mice gain more body mass. Line graphs showing weekly change in body mass in male (*n* = 9/group) and female (*n* = 9/group) Crhr2^+/−^ mice on chow and HFD. Data is presented as change in body mass per week compared to baseline. (**a**) Crhr2^+/−^ male mice gained ~ 18.6% body mass on chow and ~ 45% on HFD. (**b**): Bar graphs showing percent (%) change in body mass for chow- or HFD-fed mice. HFD-fed Crhr2^+/−^ male mice gained ~ 26% more body mass than chow-fed Crhr2^+/−^ mice. (**c**) Crhr2^+/−^ female mice gained ~ 8.8% body mass on chow and nearly ~ 50.5% on HFD. (**d**) HFD-fed Crhr2^+/−^ female mice gained ~ 41.7% more body mass than chow-fed Crhr2^+/−^ mice. 3-Way ANOVA and post hoc Tukey’s multiple comparisons as described in main text and figure legends. (DOCX 224 kb)

Additional file 2: Weekly food intake in mice. Column bar graphs showing weekly average food intake per mouse in g/ g body weight. (**a**) Crhr2^−/−^ mice increased chow intake by 25.71% and 36.89% compared with WT and Crhr2^+/−^ mice (*n* = 8/group) (**b**) WT female mice consumed 27.57% more HFD per week than Crhr2 null littermates (*n* = 8/group). 3-Way ANOVA and post hoc Tukey’s multiple comparisons. (DOCX 183 kb)

Additional file 3: Crhr2^+/−^ male mice have elevated baseline blood glucose levels. In GTT, blood glucose was measured by tail vein sampling before glucose administration (baseline; 0 min) and at 30, 60, and 120 min after a bolus of intraperitoneal glucose (2 g/kg) injection. Repeated-measure ANOVA followed by Sidak’s post hoc test was used to analyze GTT data. (**a**) In Crhr2^+/−^ male mice, blood glucose levels were significantly elevated at 0, 30 and 60 min time points on HFD vs. chow. Glucose clearance rate, as reflected by AUC, was not significantly different on HFD- vs. chow. (**b**) In female Crhr2^+/−^ HFD-fed mice, significant increases in blood glucose levels were noted at 30 only post glucose injection and AUC was higher in HFD-fed vs. chow-fed female mice. (*n* = 8/group/sex). (DOCX 259 kb)

Additional file 4: Blood glucose levels in response to insulin challenge in Crhr2^+/−^ mice. In ITT, blood glucose levels were measured by tail-vein sampling before (baseline; 0 min) and at 15, 30, 45, and 60 min after ip insulin administration in chow- and HFD-fed mice. Repeated-measure ANOVA followed by Sidak’s post hoc test was used to analyze ITT data. (**a-b**) Male and female Crhr2^+/−^ mice retained normal ITT responses on chow and HFD. *n* = 8/group/sex. (DOCX 282 kb)

Additional file 5: Male mice have increased plasma insulin levels on HFD. Column graphs showing plasma insulin levels in chow- and HFD-fed male and female mice. (**a**) Significant increases in insulin levels were seen in male WT, Crhr2^−/−^, and Crhr2^+/−^ mice (HFD vs. chow, *n* = 9/group). (**b**) HFD intake did not significantly increase plasma insulin levels in female WT, Crhr2^−/−^, and Crhr2^+/−^ mice (*n* = 9/group). Three-way ANOVA and post hoc Tukey’s multiple comparisons. (DOCX 310 kb)

Additional file 6: Sex-specific fat mass gain and/or redistribution on chow vs. HFD. Column bar graphs showing fat mass gain over 8 weeks on chow vs. HFD consumption in male and female mice. Four types of fat mass—gonadal (epididymal/ovarian), mesenteric, perirenal, and brown—were assessed. (**a**) Diet did not change gonadal fat depots in WT and Crhr2^−/−^ male mice, whereas Crhr2^+/−^ mice gained 75.72% more epididymal fat vs. chow. (**b**) HFD-fed WT and Crhr2^−/−^ male mice increased their mesenteric fat depots by 53.79% and 145.75%, respectively compared with WT chow-fed controls, whereas Crhr2+/− mice showed non-significant increases in mesenteric fat depots vs. chow. (**c**) HFD-fed WT male mice increased their perirenal fat depots by 32.67% and male Crhr2^+/−^ mice gained 70.68% more perirenal fat mass vs. chow. (**d**) HFD-fed male Crhr2^−/−^ mice increased their brown fat depots by 253.1% compared with chow-fed WT mice. (**e**) HFD-fed female Crhr2^−/−^ mice gained 103.91% more ovarian fat vs. chow diet. Female Crhr2^+/−^ mice did not show any significant change in fat mass on HFD compared with any other group. (**f**) HFD-fed female Crhr2^−/−^ mice increased their mesenteric fat depots by 38% vs. chow. (**g**) HFD-fed female Crhr2^−/−^ mice increased their perirenal fat mass by 140% vs. chow diet and by ~ 59% compared with HFD-fed WT female mice. (**h**) HFD-fed female Crhr2^−/−^ mice increased their brown fat depots by 60.86% vs. chow controls. *n* = 5/group/sex. Three-way ANOVA and post hoc Tukey’s multiple comparisons. (DOCX 530 kb)

Additional file 7: Male mice have increased plasma lipid levels. Column bar graphs showing plasma lipid profiles in male and female mice. Plasma cholesterol, HDL, triglycerides, and LDL levels were determined. (**a**) HFD-fed male WT mice had 37.7%, Crhr2^+/−^ mice had 50.3%, and Crhr2^−/−^ mice had 40.9% higher blood cholesterol levels vs. chow diet. (**b**) In female WT, Crhr2^+/−^, and Crhr2^−/−^ mice, HFD consumption resulted in smaller, non-significant increases in blood cholesterol levels vs. chow. (**c**) HFD-fed male WT mice had 36.1% higher and Crhr2^+/−^ had 38.3% higher HDL levels vs. chow. (**d**) In female mice, HDL levels did not differ between HFD vs. chow. (**e**) HFD-fed male Crhr2^−/−^ mice had ~ 53.0% higher calculated LDL levels vs. chow-fed Crhr2^−/−^ and HFD-fed WT mice, whereas Crhr2^+/−^ had 68.0% higher LDL vs. chow. (**f**) In female mice, diet did not change LDL levels. (**g**) In male mice, diet did not change triglycerides levels. (**h**) Female Crhr2^+/−^ mice had significantly elevated triglyceride levels on both chow and HFD compared with WT and Crhr2^−/−^ female mice on chow and on HFD vs. WT chow. *n* = 8/group/sex. 3-Way ANOVA and post hoc Tukey’s multiple comparisons. (DOCX 524 kb)

## Abbreviations

CRF_2_: Corticotropin-releasing factor receptor 2
Crhr2: Gene name for CRF_2_
GTT: Glucose tolerance test
HDL: High-density lipoprotein
HFD: High-fat diet
HPA axis: Hypothalamic-pituitary-adrenal axis
ITT: Insulin tolerance test
LDL: Low-density lipoprotein
UCN2 and 3: Urocortin 2 and 3
WT: Wild-type
