# Supplementary material for: Sexually dimorphic metabolic responses mediated by CRF2 receptor during nutritional stress in mice
Source: Biol Sex Differ. 2018 Nov 6;9:49. doi: 10.1186/s13293-018-0208-4 (PMC6218963; doi:10.1186/s13293-018-0208-4)
Supplement: Supplementary file 1 — Crhr2+/− male mice gain more body mass. Line graphs showing weekly change in body mass in male (n = 9/group) and female (n = 9/group) Crhr2+/− mice on chow and HFD. Data is presented as change in body mass per week compared to baseline. (a) Crhr2+/− male mice gained ~ 18.6% body mass on chow and ~ 45% on HFD. (b): Bar graphs showing percent (%) change in body mass for chow- or HFD-fed mice. HFD-fed Crhr2+/− male mice gained ~ 26% more body mass than chow-fed Crhr2+/− mice. (c) Crhr2+/− female mice gained ~ 8.8% body mass on chow and nearly ~ 50.5% on HFD. (d) HFD-fed Crhr2+/− female mice gained ~ 41.7% more body mass than chow-fed Crhr2+/− mice. 3-Way ANOVA and post hoc Tukey’s multiple comparisons as described in main text and figure legends. (DOCX 224 kb) [file 13293_2018_208_MOESM1_ESM.docx]

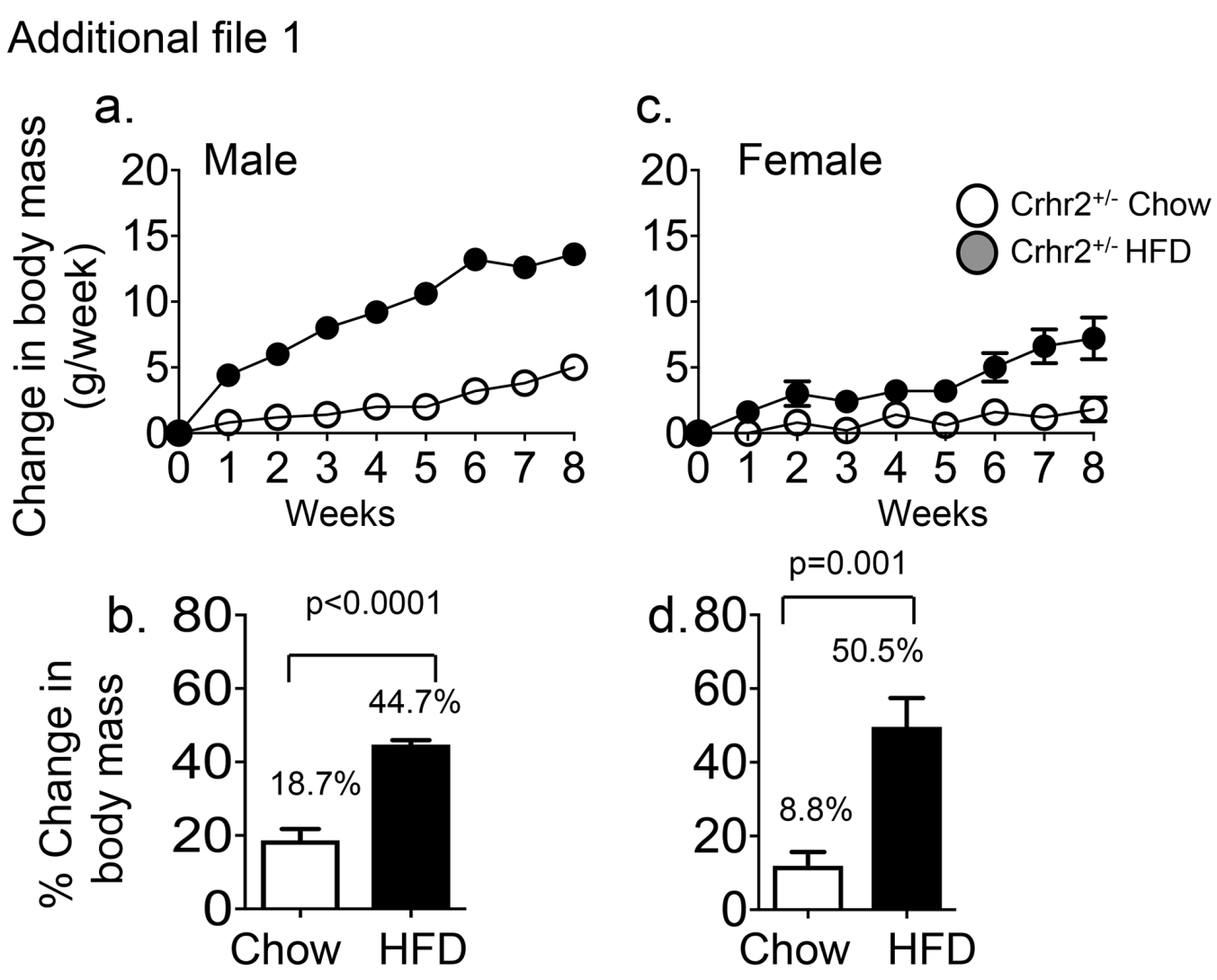


**Additional File 1 legend. Crhr2^+/-^ male mice gain more body mass**. Line graphs showing weekly change in body mass in male (n=9/group) and female (n=9/group) Crhr2^+/-^ mice on chow and HFD. Data is presented as change in body mass per week compared to baseline. (**a**) Crhr2^+/-^ male mice gained ~ 18.6% body mass on chow and ~45% on HFD. (**b**): Bar graphs showing percent (%) change in body mass for chow- or HFD-fed mice. HFD-fed Crhr2^+/-^ male mice gained ~26% more body mass than chow-fed Crhr2^+/-^ mice. (**c**) Crhr2^+/-^ female mice gained ~ 8.8% body mass on chow and nearly ~50.5% on HFD. (**d**) HFD-fed Crhr2^+/-^ female mice gained ~41.7% more body mass than chow-fed Crhr2^+/-^ mice. 3-Way ANOVA and post hoc Tukey’s multiple comparisons as described in main text and figure legends.
