# Supplementary material for: Sexually dimorphic metabolic responses mediated by CRF2 receptor during nutritional stress in mice
Source: Biol Sex Differ. 2018 Nov 6;9:49. doi: 10.1186/s13293-018-0208-4 (PMC6218963; doi:10.1186/s13293-018-0208-4)
Supplement: Supplementary file 2 — Weekly food intake in mice. Column bar graphs showing weekly average food intake per mouse in g/ g body weight. (a) Crhr2−/− mice increased chow intake by 25.71% and 36.89% compared with WT and Crhr2+/− mice (n = 8/group) (b) WT female mice consumed 27.57% more HFD per week than Crhr2 null littermates (n = 8/group). 3-Way ANOVA and post hoc Tukey’s multiple comparisons. (DOCX 183 kb) [file 13293_2018_208_MOESM2_ESM.docx]

**
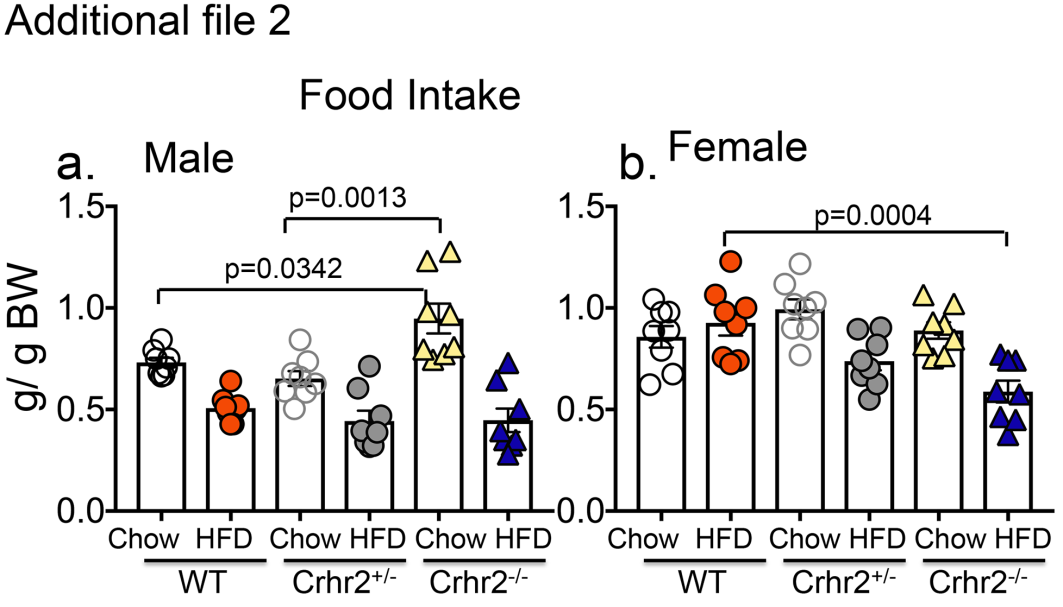
**

**Additional File 2 legend. Weekly food intake in mice**. Column bar graphs showing weekly average food intake per mouse in g/ g body weight. (**a**) Crhr2^-/-^ mice increased chow intake by 25.71% and 36.89 % compared with WT and Crhr2^+/-^ mice (n=8/group) (**b**) WT female mice consumed 27.57% more HFD per week than Crhr2 null littermates (n=8/group). 3-Way ANOVA and post hoc Tukey’s multiple comparisons.
