# Supplementary material for: Sexually dimorphic metabolic responses mediated by CRF2 receptor during nutritional stress in mice
Source: Biol Sex Differ. 2018 Nov 6;9:49. doi: 10.1186/s13293-018-0208-4 (PMC6218963; doi:10.1186/s13293-018-0208-4)
Supplement: Supplementary file 3 — Crhr2+/− male mice have elevated baseline blood glucose levels. In GTT, blood glucose was measured by tail vein sampling before glucose administration (baseline; 0 min) and at 30, 60, and 120 min after a bolus of intraperitoneal glucose (2 g/kg) injection. Repeated-measure ANOVA followed by Sidak’s post hoc test was used to analyze GTT data. (a) In Crhr2+/− male mice, blood glucose levels were significantly elevated at 0, 30 and 60 min time points on HFD vs. chow. Glucose clearance rate, as reflected by AUC, was not significantly different on HFD- vs. chow. (b) In female Crhr2+/− HFD-fed mice, significant increases in blood glucose levels were noted at 30 only post glucose injection and AUC was higher in HFD-fed vs. chow-fed female mice. (n = 8/group/sex). (DOCX 259 kb) [file 13293_2018_208_MOESM3_ESM.docx]

**
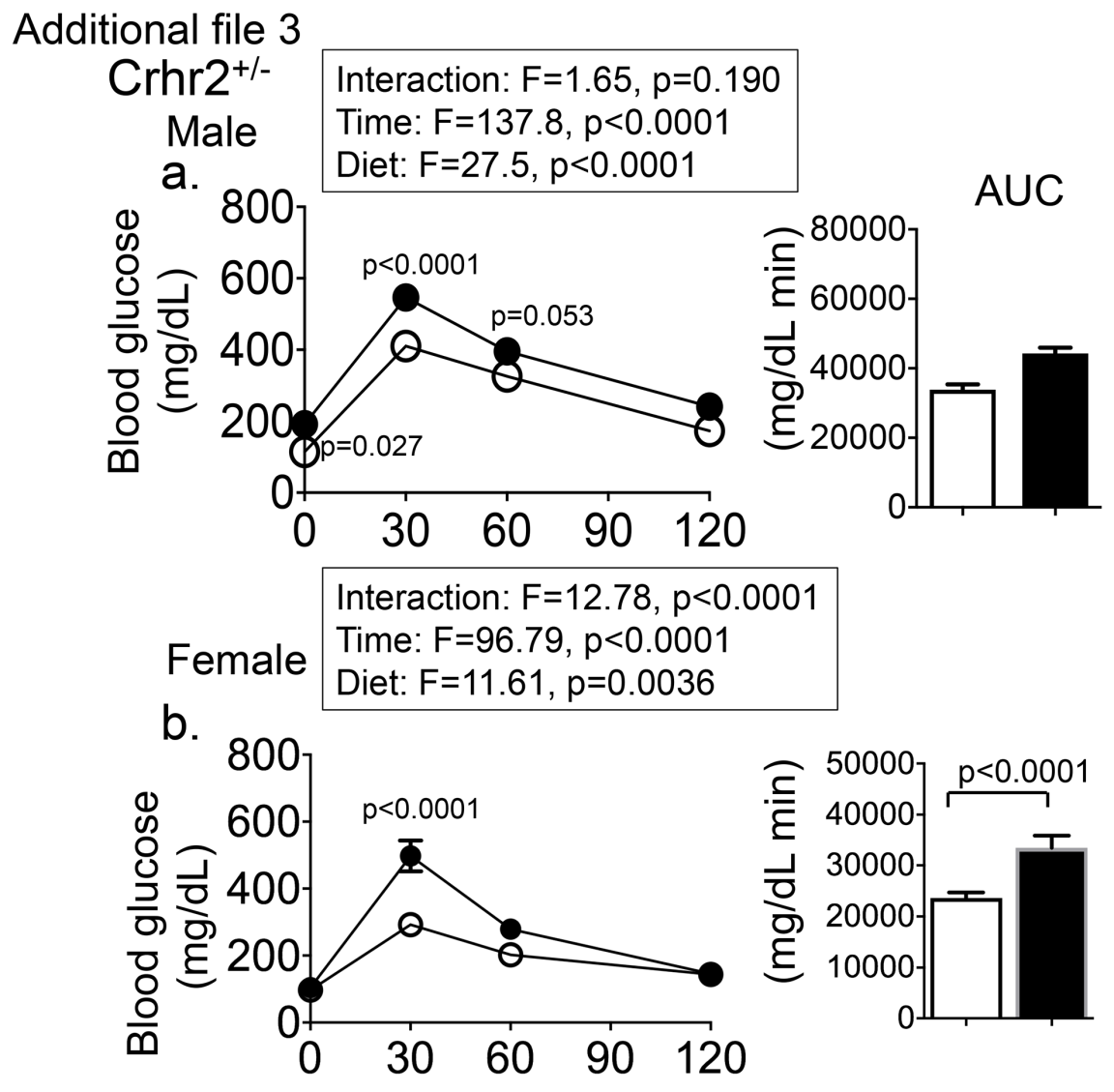
**

**Additional File 3 legend. Crhr2^+/-^ male mice have elevated baseline** **blood glucose levels**. In GTT, blood glucose was measured by tail vein sampling before glucose administration (baseline; 0 min) and at 30, 60, and 120 minutes after a bolus of intraperitoneal glucose (2g/kg) injection. Repeated-measure ANOVA followed by Sidak’s *post hoc* test was used to analyze GTT data. (**a**) In Crhr2^+/-^ male mice, blood glucose levels were significantly elevated at 0, 30 and 60 min time points on HFD vs. chow. Glucose clearance rate, as reflected by AUC, was not significantly different on HFD- vs. chow. (**b**) In female Crhr2^+/-^ HFD-fed mice, significant increases in blood glucose levels were noted at 30 only post glucose injection and AUC was higher in HFD-fed vs. chow-fed female mice. (n=8/group/sex).
