# Supplementary material for: Sexually dimorphic metabolic responses mediated by CRF2 receptor during nutritional stress in mice
Source: Biol Sex Differ. 2018 Nov 6;9:49. doi: 10.1186/s13293-018-0208-4 (PMC6218963; doi:10.1186/s13293-018-0208-4)
Supplement: Supplementary file 4 — Blood glucose levels in response to insulin challenge in Crhr2+/− mice. In ITT, blood glucose levels were measured by tail-vein sampling before (baseline; 0 min) and at 15, 30, 45, and 60 min after ip insulin administration in chow- and HFD-fed mice. Repeated-measure ANOVA followed by Sidak’s post hoc test was used to analyze ITT data. (a-b) Male and female Crhr2+/− mice retained normal ITT responses on chow and HFD. n = 8/group/sex. (DOCX 282 kb) [file 13293_2018_208_MOESM4_ESM.docx]

**
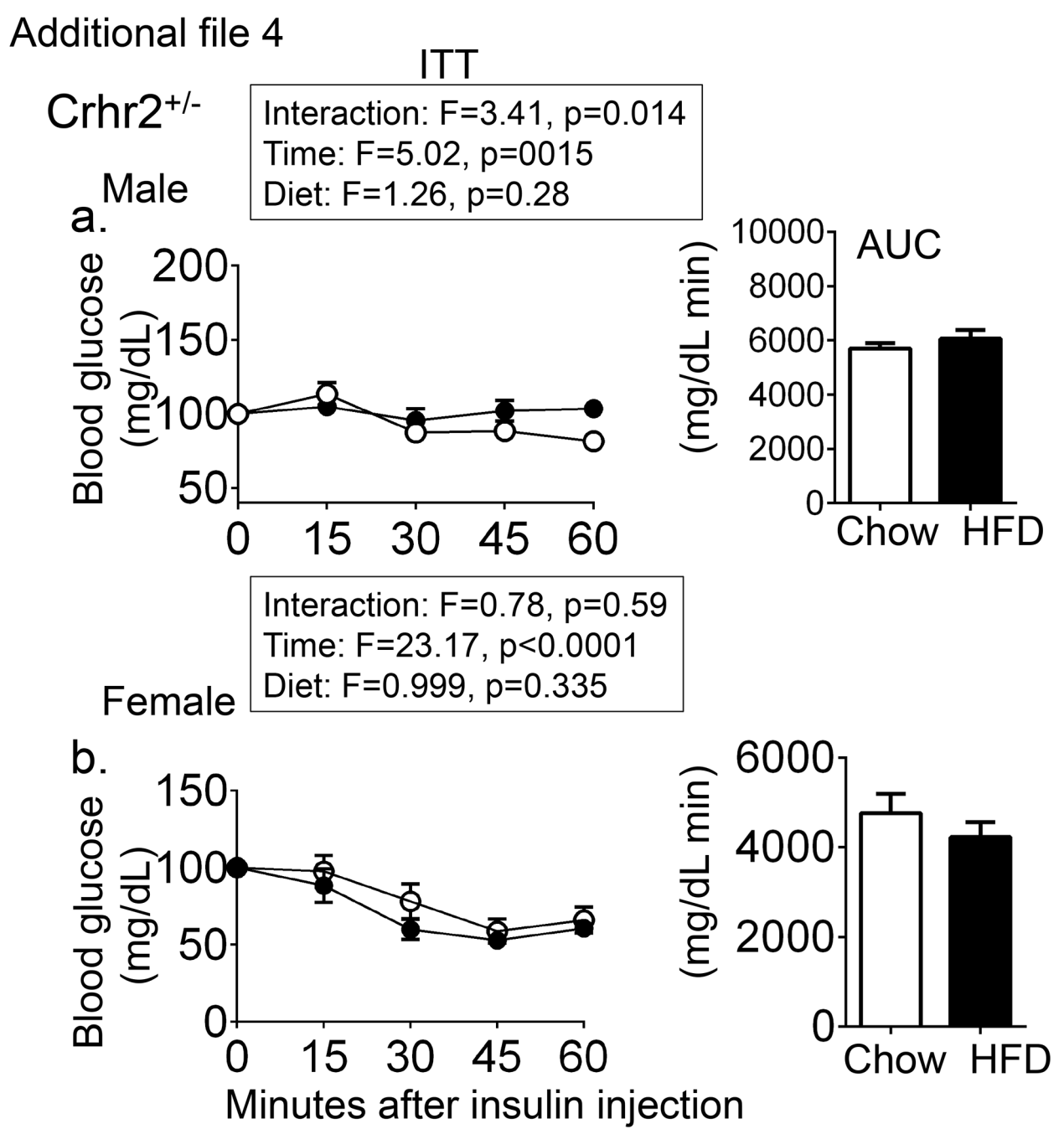
**

**Additional File 4 legend. Blood glucose levels in response to insulin challenge in Crhr2^+/-^ mice**. In ITT, blood glucose levels were measured by tail-vein sampling before (baseline; 0 min) and at 15, 30, 45 and 60 minutes after ip insulin administration in chow- and HFD-fed mice. Repeated-measure ANOVA followed by Sidak’s *post hoc* test was used to analyze ITT data. (**a-b**) Male and female Crhr2^+/-^ mice retained normal ITT responses on chow and HFD. n= 8/group/sex.
