# Supplementary material for: Sexually dimorphic metabolic responses mediated by CRF2 receptor during nutritional stress in mice
Source: Biol Sex Differ. 2018 Nov 6;9:49. doi: 10.1186/s13293-018-0208-4 (PMC6218963; doi:10.1186/s13293-018-0208-4)
Supplement: Supplementary file 5 — Male mice have increased plasma insulin levels on HFD. Column graphs showing plasma insulin levels in chow- and HFD-fed male and female mice. (a) Significant increases in insulin levels were seen in male WT, Crhr2−/−, and Crhr2+/− mice (HFD vs. chow, n = 9/group). (b) HFD intake did not significantly increase plasma insulin levels in female WT, Crhr2−/−, and Crhr2+/− mice (n = 9/group). Three-way ANOVA and post hoc Tukey’s multiple comparisons. (DOCX 310 kb) [file 13293_2018_208_MOESM5_ESM.docx]

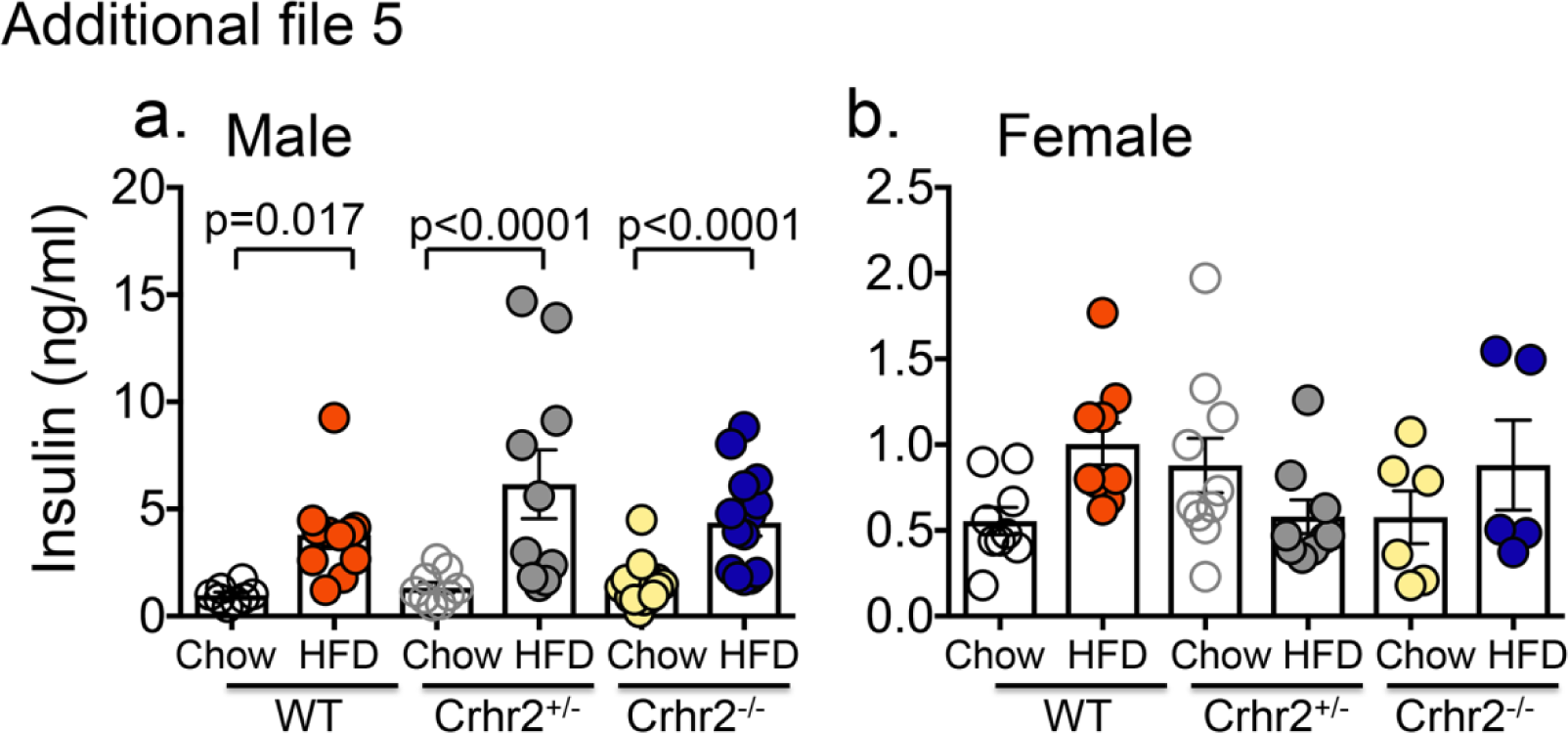


**Additional File 5 legend. Male mice have increased plasma insulin levels on HFD**. Column graphs showing plasma insulin levels in chow- and HFD-fed male and female mice. (**a**) Significant increases in insulin levels were seen in male WT, Crhr2^-/-^, and Crhr2^+/-^ mice (HFD vs. chow, n= 9/group). (**b**) HFD intake did not significantly increase plasma insulin levels in female WT, Crhr2^-/-^, and Crhr2^+/-^ mice (n=9/group). 3-Way ANOVA and post hoc Tukey’s multiple comparisons.
