# Supplementary material for: Sexually dimorphic metabolic responses mediated by CRF2 receptor during nutritional stress in mice
Source: Biol Sex Differ. 2018 Nov 6;9:49. doi: 10.1186/s13293-018-0208-4 (PMC6218963; doi:10.1186/s13293-018-0208-4)
Supplement: Supplementary file 6 — Sex-specific fat mass gain and/or redistribution on chow vs. HFD. Column bar graphs showing fat mass gain over 8 weeks on chow vs. HFD consumption in male and female mice. Four types of fat mass—gonadal (epididymal/ovarian), mesenteric, perirenal, and brown—were assessed. (a) Diet did not change gonadal fat depots in WT and Crhr2−/− male mice, whereas Crhr2+/− mice gained 75.72% more epididymal fat vs. chow. (b) HFD-fed WT and Crhr2−/− male mice increased their mesenteric fat depots by 53.79% and 145.75%, respectively compared with WT chow-fed controls, whereas Crhr2+/− mice showed non-significant increases in mesenteric fat depots vs. chow. (c) HFD-fed WT male mice increased their perirenal fat depots by 32.67% and male Crhr2+/− mice gained 70.68% more perirenal fat mass vs. chow. (d) HFD-fed male Crhr2−/− mice increased their brown fat depots by 253.1% compared with chow-fed WT mice. (e) HFD-fed female Crhr2−/− mice gained 103.91% more ovarian fat vs. chow diet. Female Crhr2+/− mice did not show any significant change in fat mass on HFD compared with any other group. (f) HFD-fed female Crhr2−/− mice increased their mesenteric fat depots by 38% vs. chow. (g) HFD-fed female Crhr2−/− mice increased their perirenal fat mass by 140% vs. chow diet and by ~ 59% compared with HFD-fed WT female mice. (h) HFD-fed female Crhr2−/− mice increased their brown fat depots by 60.86% vs. chow controls. n = 5/group/sex. Three-way ANOVA and post hoc Tukey’s multiple comparisons. (DOCX 530 kb) [file 13293_2018_208_MOESM6_ESM.docx]

**
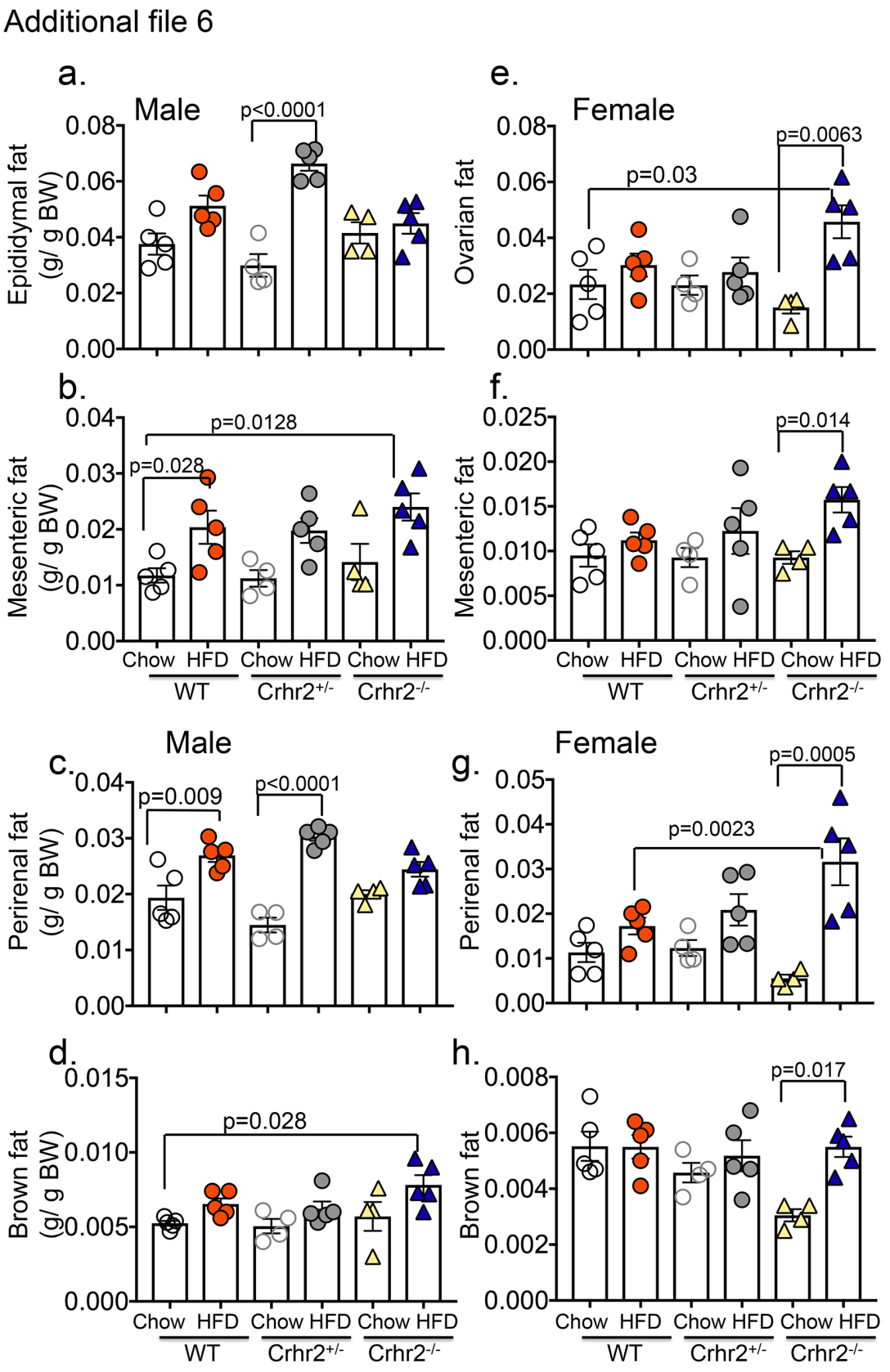
**

**Additional File 6 legend. Sex-specific fat mass gain and/or redistribution on chow vs. HFD**. Column bar graphs showing fat mass gain over 8 weeks on chow vs. HFD consumption in male and female mice. Four types of fat mass- gonadal (epididymal/ovarian), mesenteric, perirenal, and brown were assessed. (**a**) Diet did not change gonadal fat depots in WT and Crhr2^-/-^ male mice, whereas Crhr2^+/-^ mice gained 75.72% more epididymal fat vs. chow. (**b**) HFD-fed WT and Crhr2^-/-^ male mice increased their mesenteric fat depots by 53.79% and 145.75%, respectively compared with WT chow-fed controls, whereas Crhr2+/- mice showed non-significant increases in mesenteric fat depots vs. chow. (**c**) HFD-fed WT male mice increased their perirenal fat depots by 32.67% and male Crhr2^+/-^ mice gained 70.68% more perirenal fat mass vs. chow. (**d**) HFD-fed male Crhr2^-/-^ mice increased their brown fat depots by 253.1% compared with chow-fed WT mice. (**e**) HFD-fed female Crhr2^-/-^ mice gained 103.91% more ovarian fat vs. chow diet. Female Crhr2^+/-^ mice did not show any significant change in fat mass on HFD compared with any other group. (**f**) HFD-fed female Crhr2^-/-^ mice increased their mesenteric fat depots by 38% vs. chow. (**g**) HFD-fed female Crhr2^-/-^ mice increased their perirenal fat mass by 140% vs. chow diet and by ~59% compared with HFD-fed WT female mice. (**h**) HFD-fed female Crhr2^-/-^ mice increased their brown fat depots by 60.86% vs. chow controls. n=5/group/sex. 3-Way ANOVA and post hoc Tukey’s multiple comparisons.
