# Supplementary material for: Sexually dimorphic metabolic responses mediated by CRF2 receptor during nutritional stress in mice
Source: Biol Sex Differ. 2018 Nov 6;9:49. doi: 10.1186/s13293-018-0208-4 (PMC6218963; doi:10.1186/s13293-018-0208-4)
Supplement: Supplementary file 7 — Male mice have increased plasma lipid levels. Column bar graphs showing plasma lipid profiles in male and female mice. Plasma cholesterol, HDL, triglycerides, and LDL levels were determined. (a) HFD-fed male WT mice had 37.7%, Crhr2+/− mice had 50.3%, and Crhr2−/− mice had 40.9% higher blood cholesterol levels vs. chow diet. (b) In female WT, Crhr2+/−, and Crhr2−/− mice, HFD consumption resulted in smaller, non-significant increases in blood cholesterol levels vs. chow. (c) HFD-fed male WT mice had 36.1% higher and Crhr2+/− had 38.3% higher HDL levels vs. chow. (d) In female mice, HDL levels did not differ between HFD vs. chow. (e) HFD-fed male Crhr2−/− mice had ~ 53.0% higher calculated LDL levels vs. chow-fed Crhr2−/− and HFD-fed WT mice, whereas Crhr2+/− had 68.0% higher LDL vs. chow. (f) In female mice, diet did not change LDL levels. (g) In male mice, diet did not change triglycerides levels. (h) Female Crhr2+/− mice had significantly elevated triglyceride levels on both chow and HFD compared with WT and Crhr2−/− female mice on chow and on HFD vs. WT chow. n = 8/group/sex. 3-Way ANOVA and post hoc Tukey’s multiple comparisons. (DOCX 524 kb) [file 13293_2018_208_MOESM7_ESM.docx]

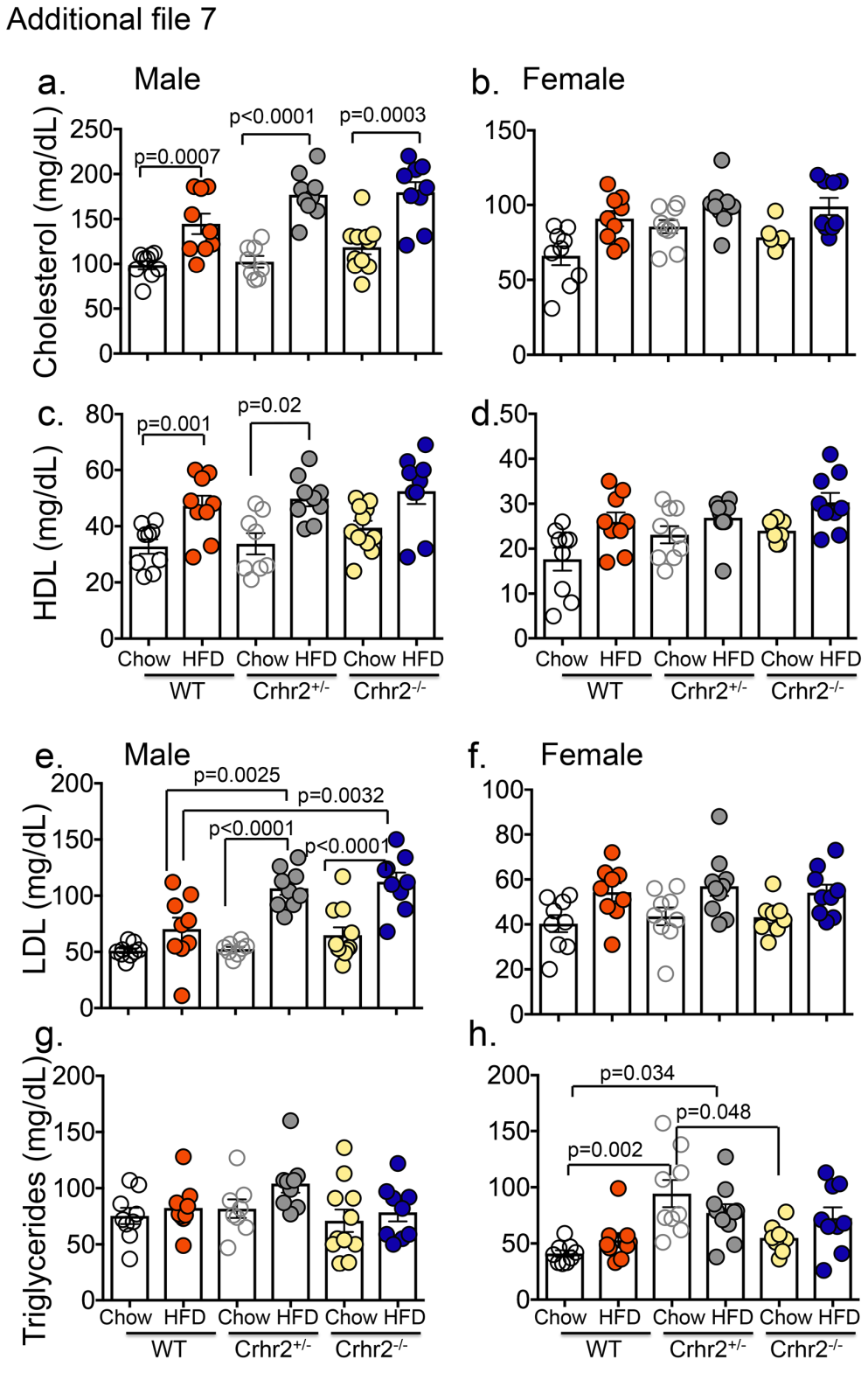


**Additional File 7 legend. Male mice have increased plasma lipid levels**. Column bar graphs showing plasma lipid profiles in male and female mice. Plasma cholesterol, HDL, triglycerides, and LDL levels were determined. (**a**) HFD-fed male WT mice had 37.7%, Crhr2^+/-^ mice had 50.3%, and Crhr2^-/-^ mice had 40.9% higher blood cholesterol levels vs. chow diet. (**b**) In female WT, Crhr2^+/-^, and Crhr2^-/-^ mice, HFD consumption resulted in smaller, non-significant increases in blood cholesterol levels vs. chow. (**c**) HFD-fed male WT mice had 36.1% higher and Crhr2^+/-^ had 38.3% higher HDL levels vs. chow. (**d**) In female mice, HDL levels did not differ between HFD vs. chow. (**e**) HFD-fed male Crhr2^-/-^ mice had ~53.0% higher calculated LDL levels vs. chow-fed Crhr2^-/-^ and HFD-fed WT mice, whereas Crhr2^+/-^ had 68.0% higher LDL vs. chow. (**f**) In female mice, diet did not change LDL levels. (**g**) In male mice, diet did not change triglycerides levels. (**h**) Female Crhr2^+/-^ mice had significantly elevated triglyceride levels on both chow and HFD compared with WT and Crhr2^-/-^ female mice on chow and on HFD vs. WT chow. n=8/group/sex. 3-Way ANOVA and post hoc Tukey’s multiple comparisons.
